# Escalation in the host-pathogen arms race: A host resistance response corresponds to a heightened bacterial virulence response

**DOI:** 10.1371/journal.ppat.1009175

**Published:** 2021-01-11

**Authors:** Qi Wang, Nadia Shakoor, Adam Boyher, Kira M. Veley, Jeffrey C. Berry, Todd C. Mockler, Rebecca S. Bart

**Affiliations:** Donald Danforth Plant Science Center, Saint Louis, Missouri, United States of America; The Ohio State University, UNITED STATES

## Abstract

The zig-zag model of host-pathogen interaction describes the relative strength of defense response across a spectrum of pathogen-induced plant phenotypes. A stronger defense response results in increased resistance. Here, we investigate the strength of pathogen virulence during disease and place these findings in the context of the zig-zag model. *Xanthomonas vasicola* pv. *holcicola* (*Xvh*) causes sorghum bacterial leaf streak. Despite being widespread, this disease has not been described in detail at the molecular level. We divided diverse sorghum genotypes into three groups based on disease symptoms: water-soaked lesions, red lesions, and resistance. Bacterial growth assays confirmed that these three phenotypes represent a range of resistance and susceptibility. To simultaneously reveal defense and virulence responses across the spectrum of disease phenotypes, we performed dual RNA-seq on *Xvh*-infected sorghum. Consistent with the zig-zag model, the expression of plant defense-related genes was strongest in the resistance interaction. Surprisingly, bacterial virulence genes related to the type III secretion system (T3SS) and type III effectors (T3Es) were also most highly expressed in the resistance interaction. This expression pattern was observed at multiple time points within the sorghum-*Xvh* pathosystem. Further, a similar expression pattern was observed in *Arabidopsis* infected with *Pseudomonas syringae* for effector-triggered immunity via AvrRps4 but not AvrRpt2. Specific metabolites were able to repress the *Xvh* virulence response *in vitro* and *in planta* suggesting a possible signaling mechanism. Taken together, these findings reveal multiple permutations of the continually evolving host-pathogen arms race from the perspective of host defense and pathogen virulence responses.

## Author summary

The arms race between plants and pathogens is a complex process. To dissect the plant defense and pathogen virulence responses simultaneously, we used sorghum and *Xanthomonas vasicola* pv. *holcicola*, as a model pathosystem. We performed dual RNA-seq on infected sorghum with a range of disease phenotypes. Our characterization of this pathosystem demonstrates that genes related to the plant defense and pathogen virulence responses are most highly induced during a resistance interaction. We observed a similar pattern of escalation in *Arabidopsis* infected with *Pseudomonas syringae*. These observations support a conceptual model of fluidity between the different stages of plant immunity and pathogen virulence.

## Introduction

The arms race between plants and pathogens is a complex process conceptualized by the “zig-zag” model as follows: First, the plant recognizes microbe/pathogen-associated molecular patterns (MAMPs/PAMPs) such as bacterial flagella via host–pattern recognition receptors (PRRs) that trigger a basal defense response called PAMP-triggered immunity (PTI); second, a successful pathogen delivers effector proteins to the host to repress PTI and induce susceptibility. This is called effector-triggered susceptibility (ETS); third, the effector is recognized, directly or indirectly, by a host resistance (R) protein resulting in effector-triggered immunity (ETI) [1]. ETI occurs faster, is more prolonged and more robust than PTI. On the pathogen side, the strength of virulence across PTI, ETS, and ETI is unclear. In this study, we investigate the amplitude of this additional dimension of the zig-zag model, using a sorghum-*Xanthomonas* pathosystem.

Sorghum [*Sorghum bicolor* (L.) Moench] is an important cereal crop used for food, animal feed, and biofuel production [2]. In semiarid and arid regions where the growth of other crops is restricted, sorghum has become a major cereal crop for human consumption [3]. The crop originated from tropical Africa and has undergone a remarkable diversification resulting in five major races and thousands of genotypes [4]. Sorghum grows across all major global agricultural production regions and is especially prized for its tolerance to abiotic stresses [5,6]. However, disease and pests limit sorghum productivity and these stresses may be exacerbated by global climate change and unintended consequences of breeding efforts [5,7].

Xanthomonads are gram-negative, motile bacteria that cause a number of agriculturally-relevant diseases [8]. *Xanthomonas*-incited diseases include rice bacterial blight, cassava bacterial blight, banana bacterial wilt, and black rot [8]. Xanthomonads are also a convenient model for studying plant-pathogen interactions because they are easy to culture, have a small genome and are genetically tractable [8–10]. A major virulence strategy employed by Xanthomonads is the type III secretion system (T3SS) through which the pathogen can deliver type III effector (T3E) proteins into host cells. These effectors serve a variety of functions related to suppressing resistance and promoting susceptibility [8,11,12].

Sorghum bacterial leaf streak disease, caused by *Xanthomonas vasicola* pv. *holcicola* (*Xvh*), has a wide geographical distribution but usually only causes minor crop losses. However, under favorable environmental conditions, this disease can cause considerable leaf damage that affects sorghum production value [13,14]. Currently, there are no effective methods for control of this disease. The highly diverse sorghum germplasm may exhibit varied disease phenotypes, although these interactions have not yet been characterized. While genome sequences are available for sorghum and *Xvh* [15,16], relatively little research has been conducted on the molecular basis of sorghum-*Xvh* interactions [17,18]. Here, we conducted a large-scale screen for resistance or susceptibility to *Xvh* in diverse sorghum genotypes and identified a range of disease phenotypes. To reveal the molecular mechanisms governing the interactions between sorghum and *Xvh*, we performed dual RNA-seq on *Xvh*-infected sorghum. We uncovered defense and virulence responses across the range of disease phenotypes, simultaneously. As expected, plant defense-related genes were most highly expressed during a ‘resistance’ interaction. Bacterial genes related to virulence, specifically T3SS and T3Es were also most highly expressed in the resistance interaction. Moreover, we found that specific metabolites (e.g., pyruvate) suppressed the virulence response *in vitro* and *in planta* suggesting a possible mechanism for host-mediated T3E repression. A similar molecular pattern was observed in the Arabidopsis-*Pseudomonas syringae* pathosystem for ETI triggered via AvrRps4 but not AvrRpt2. Taken together, these data highlight that, at least in some cases, a heightened defense response correlates with heightened expression of virulence genes in the pathogen. More generally, the results reveal multiple snapshots of the continually evolving arms race from the perspective of host defense and pathogen virulence responses.

## Results

### Screening sorghum genotypes against *X*. *vasicola* pv. *holcicola* (*Xvh*)

**Fig 1:**
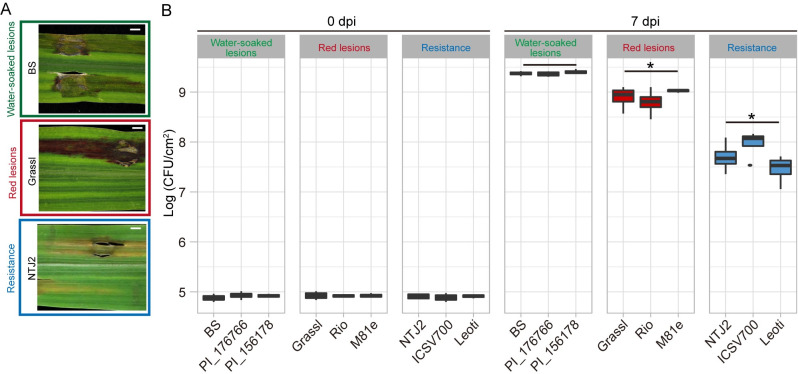
*Xvh*-disease phenotypes and bacterial growth in sorghum. (A) *Xvh* disease phenotypes: water-soaked lesions, red lesions, and resistance. (B) *In planta* bacterial growth assay. Sorghum leaves were infiltrated with *Xvh* (OD_600nm_ = 0.02 (~1 × 10^7^ cfu/mL)). Bacterial populations were determined at 0 dpi and 7 dpi and are shown as colony-forming units (CFU). The mean ± s.d. was obtained from four biological replicates. Each replicate represents two inoculation areas from one leaf on one plant. Asterisks indicate statistical significance based on unequal variances *t* test (n = 12 (three genotypes × four replicates/genotype), *p < 0.05) comparison with the water-soaked lesions phenotype. dpi, days post-inoculation.

**Fig 2:**
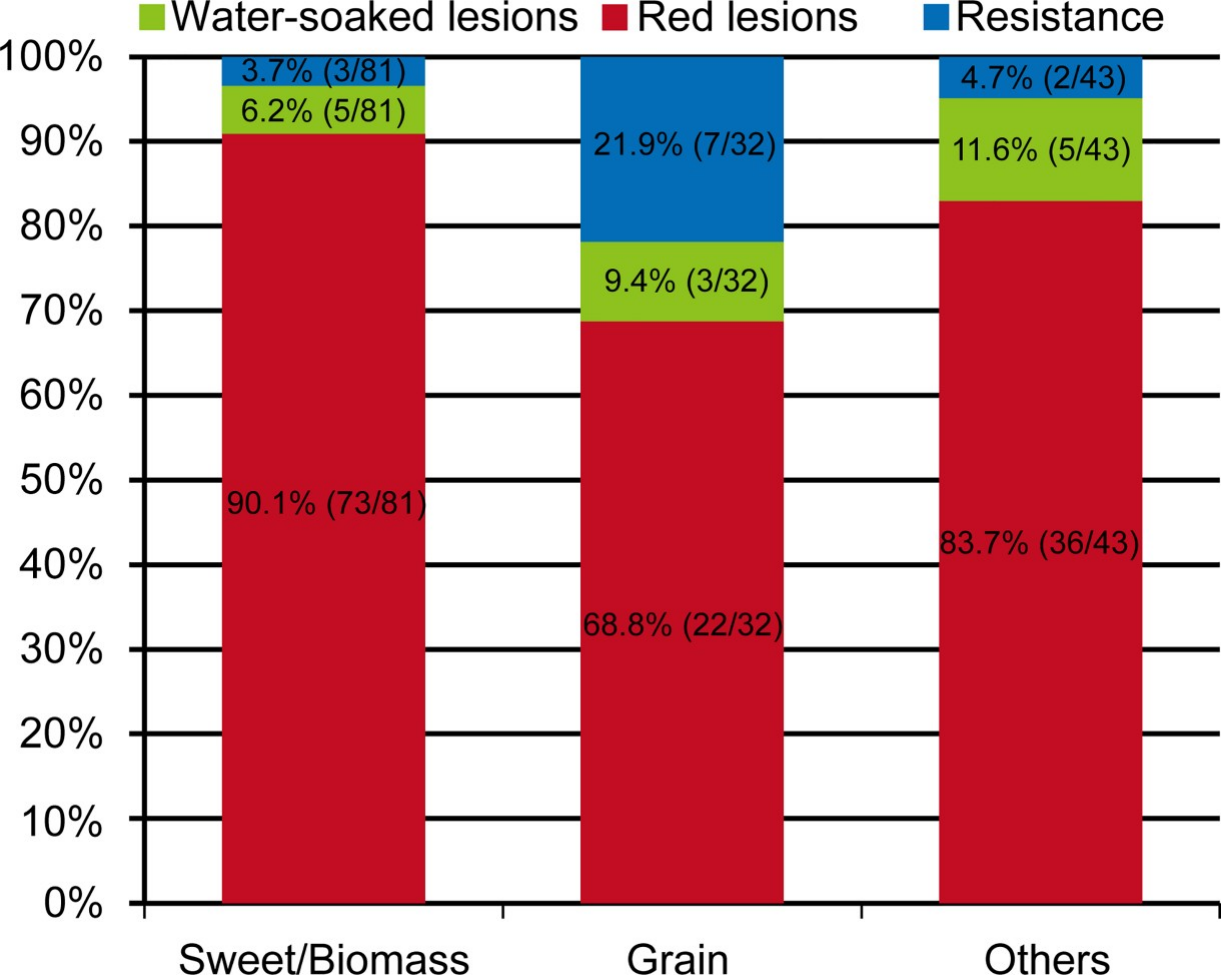
Summary of the *Xvh*-disease phenotypes in 156 sorghum genotypes. Percentage and ratio of each disease phenotype for the different types of sorghum genotypes.

To evaluate sorghum genotypes for their reaction to bacterial leaf streak disease, we screened 156 sorghum genotypes for resistance or susceptibility to the pathogen *Xvh* BLS185. These sorghum genotypes represent five basic races and ten intermediate races of sorghum (Tables 1 and S1). Each genotype was infiltrated with a bacterial suspension, and symptoms were observed 7 days post-inoculation (dpi). Among the genotypes screened, we observed three phenotypes after inoculation: water-soaked lesions, red lesions, and mild to no symptoms (resistance) (Figs 1A and S1). We did not observe a hypersensitive response or rapid cell death that, in other pathosystems, is a signature of strong resistance [19]. The most commonly observed phenotype was red lesions (84.0%). Among the sorghum genotypes that could be classified as either sweet/biomass sorghum or grain sorghum, we observed a slightly higher percentage of resistance among the latter (Figs 2 and S1 and S1 Table). To further understand the observed *Xvh* disease phenotypes, we performed bacterial growth assays on three representative genotypes for each disease phenotype. The results revealed that the water-soaked lesion phenotype observed in Black Spanish (BS), PI176766, and PI156178 supported the highest bacterial growth, followed by the red lesion phenotype observed in Grassl, Rio, and M81e. The representative genotypes that exhibited mild to no symptoms, NTJ2, ICSV700, and Leoti, in contrast, supported significantly lower bacterial populations (Figs 1 and S2). The bacterial populations in these genotypes were still higher than a nonhost pathogen *Xam668*, a *Xanthomonas* pathogen of cassava (*Manihot esculenta*) [20] (S2 Fig). Based on these data, we conclude that sorghum genotypes that develop water-soaked or red lesions are susceptible to *Xvh* while the other genotypes are resistant.

**Table 1:** *Xvh* disease phenotypes observed among diverse sorghum genotypes.

		Water-soaked lesions^†^	Red lesions^†^	Resistance^†^	Total (156)^‡^
Races*	*Bicolor*	5	23	2	30
	*Guinea*	3	9	1	13
	*Caudatum*	0	19	1	20
	*Kafir*	1	13	1	15
	*Durra*	2	14	1	17
Intermediate races*	*Guinea-bicolor*	0	3	1	4
	*Caudatum-bicolor*	0	6	1	7
	*Kafir-bicolor*	0	4	0	4
	*Durra-bicolor*	0	5	0	5
	*Guinea-caudatum*	0	6	0	6
	*Guinea-kafir*	0	1	1	2
	*Guinea-durra*	1	2	0	3
	*Kafir-caudatum*	0	4	0	4
	*Durra-caudatum*	0	3	1	4
	*Kafir-durra*	0	2	0	2
Unclassified		1	17	2	20
Percentage		8.3%	84.0%	7.7%	

*The sorghum genotypes cover five basic and ten intermediate races.

^†^Three *Xvh-*incited disease phenotypes were observed: water-soaked lesions, red lesions, and resistance. The number of genotypes displaying each phenotype is listed.

^‡^156 genotypes of sorghum were included.

**Fig 3:**
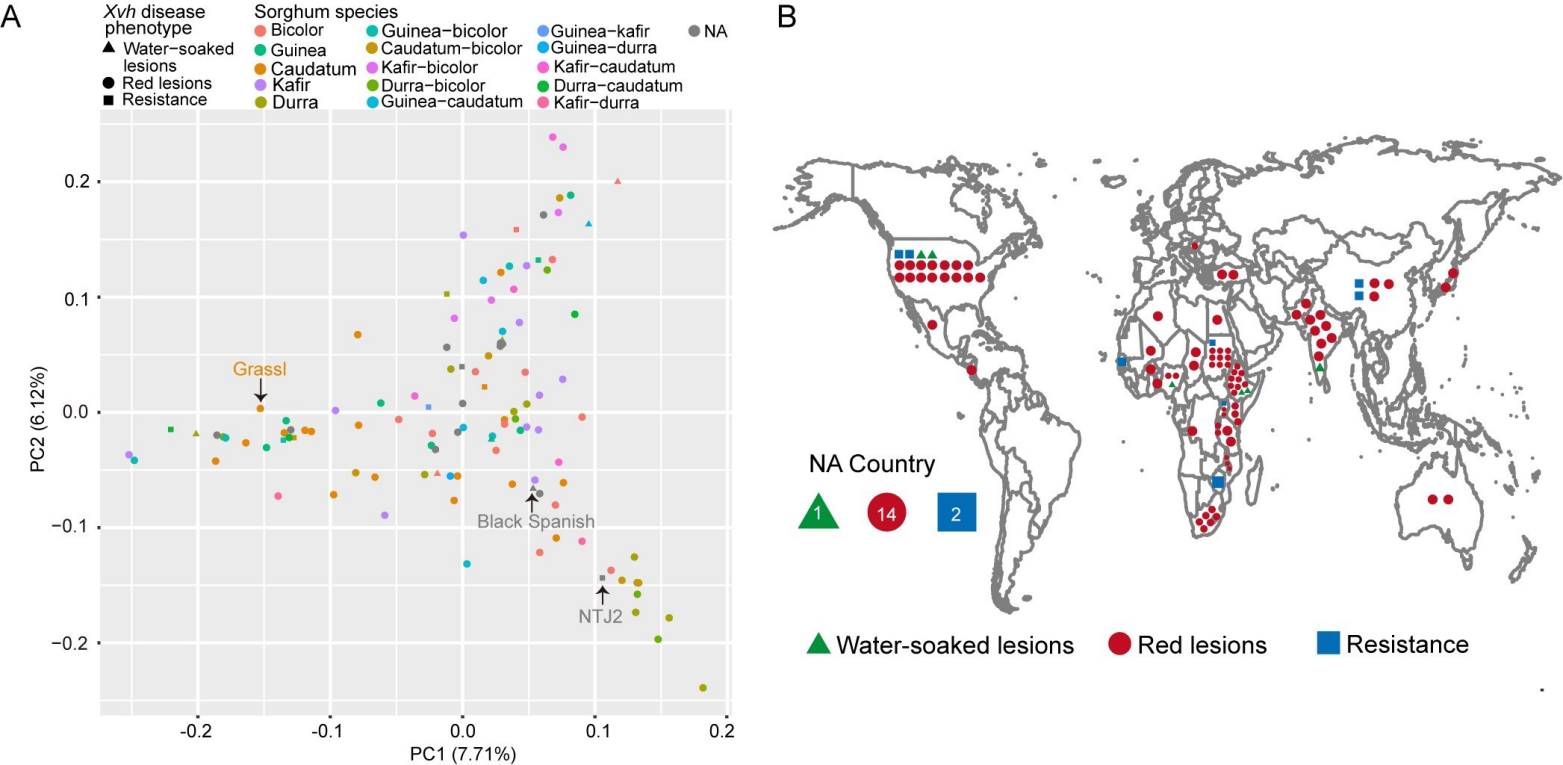
*Xvh*-disease phenotypes are distributed across 113 sorghum genotypes from diverse geographical origins. (A) Principal component analysis (PCA) of sorghum genotypes with GBS-based SNPs. Sorghum genotypes are colored based on race, and the respective disease phenotypes are indicated by symbol shape. The three *Xvh*-disease phenotypes do not cluster by PC1 or PC2. (B) Geographical distribution of sorghum genotypes. The three *Xvh*-disease phenotypes are widely distributed around the world. Black Spanish (water-soaked lesions), Grassl (red lesions), and NTJ2 (resistance).

Next, we considered whether the observed disease phenotypes correlate with sorghum genetic diversity or geographical distribution. Genotyping-by-sequencing (GBS) data is available for 113 of 156 genotypes included in this study [21] (S1 Table). These data were used to identify single nucleotide polymorphisms (SNPs) among the genotypes and to construct a phylogenetic tree. This analysis revealed that the three observed disease phenotypes did not cluster phylogenetically (S3 Fig). We note that sorghum has been significantly impacted by human intervention as genotypes have been transferred and exchanged with extensive and global breeding programs. Therefore, a neighbor joining tree may not be the most appropriate analysis. Thus, as an alternative approach, using the GBS-based SNPs, we conducted a principal component analysis (PCA) and observed that the three phenotypes are distributed across PC1 and PC2 (Fig 3). Similarly, the three phenotypes do not cluster by geographic origin (Fig 3). Together, these analyses are consistent with previous reports that this disease is common across the globe and, that historically, regional breeding programs have not prioritized resistance.

### Transcriptome analysis of sorghum-*Xvh* interaction

**Fig 4:**
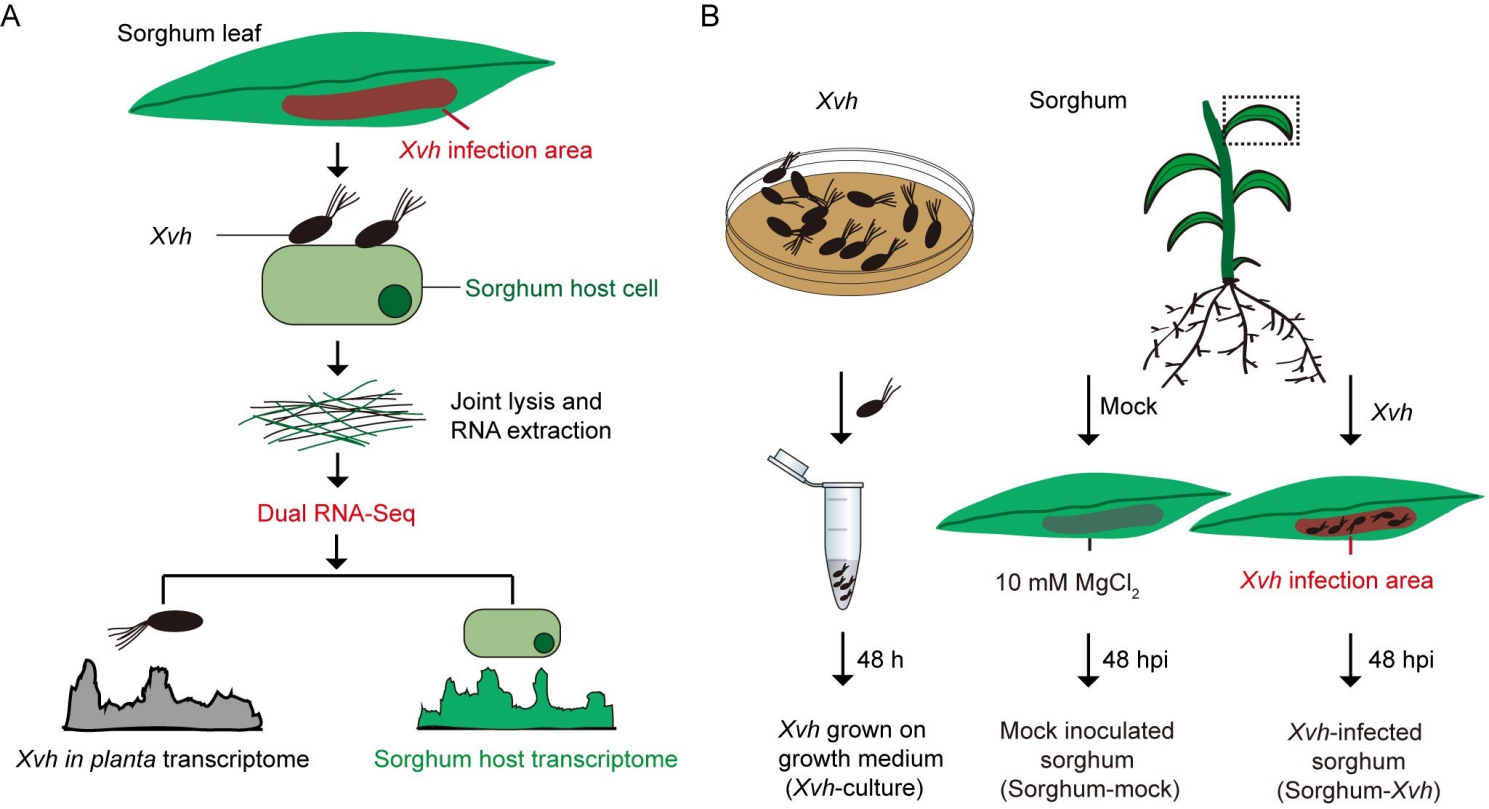
Establishment of dual RNA-Seq for transcriptional analysis of sorghum-*Xvh* interaction. (A) Workflow of dual RNA-Seq analysis (redrawn and modified from Westermann et al. 2017 [22]). (B) Schematic presentation of the sampling for dual RNA-Seq (see *Materials and Methods* for further information).

To dissect the molecular mechanisms that distinguish the three types of observed sorghum-*Xvh* interactions, we adopted a transcriptomics approach wherein we simultaneously observed gene expression changes that occur in the plant and the pathogen [22]. Sorghum leaves were inoculated with a high titer inoculum (OD_600nm_ of 0.5 (~10^9^ cfu/mL)) to achieve a unified cellular response *in planta* [23,24]. Using a high inoculum titer, there were no significant differences in the bacterial population among the three sorghum genotypes at 48 hours post-inoculation (S4 Fig). In brief, total RNA was extracted from sorghum leaves infected by *Xvh*, followed by ribosomal RNA depletion, library preparation, and RNA-sequencing (Fig 4; *Materials and Methods*). Three biological replicates for each condition (*Xvh* grown on growth medium (*Xvh*-culture), mock-inoculated sorghum (sorghum-mock: BS-mock, Grassl-mock, NTJ2-mock), and *Xvh*-infected sorghum (sorghum-*Xvh*: BS-*Xvh*, Grassl-*Xvh*, NTJ2-*Xvh*)) were sequenced. After sequencing, all reads were aligned against the BTx623 sorghum reference genome and an *Xvh* genome provided by the Joint Genome Institute (JGI) (see Materials and Methods). For the *Xvh*-culture samples, ~96% of the reads mapped to the *Xvh* genome. In the sorghum-mock samples, ~86% of the reads mapped to the BTx623 genome and an insignificant fraction mapped to the *Xvh* genome. In the sorghum-*Xvh* samples, ~78% of the reads mapped to the BTx623 genome and 1–5% mapped to the *Xvh* genome (S2 Table). For subsequent analysis, gene expression values were calculated using only the reads that mapped to the *Xvh* genome or the BTx623 genome, for *Xvh* or sorghum genes, respectively.

**Fig 5:**
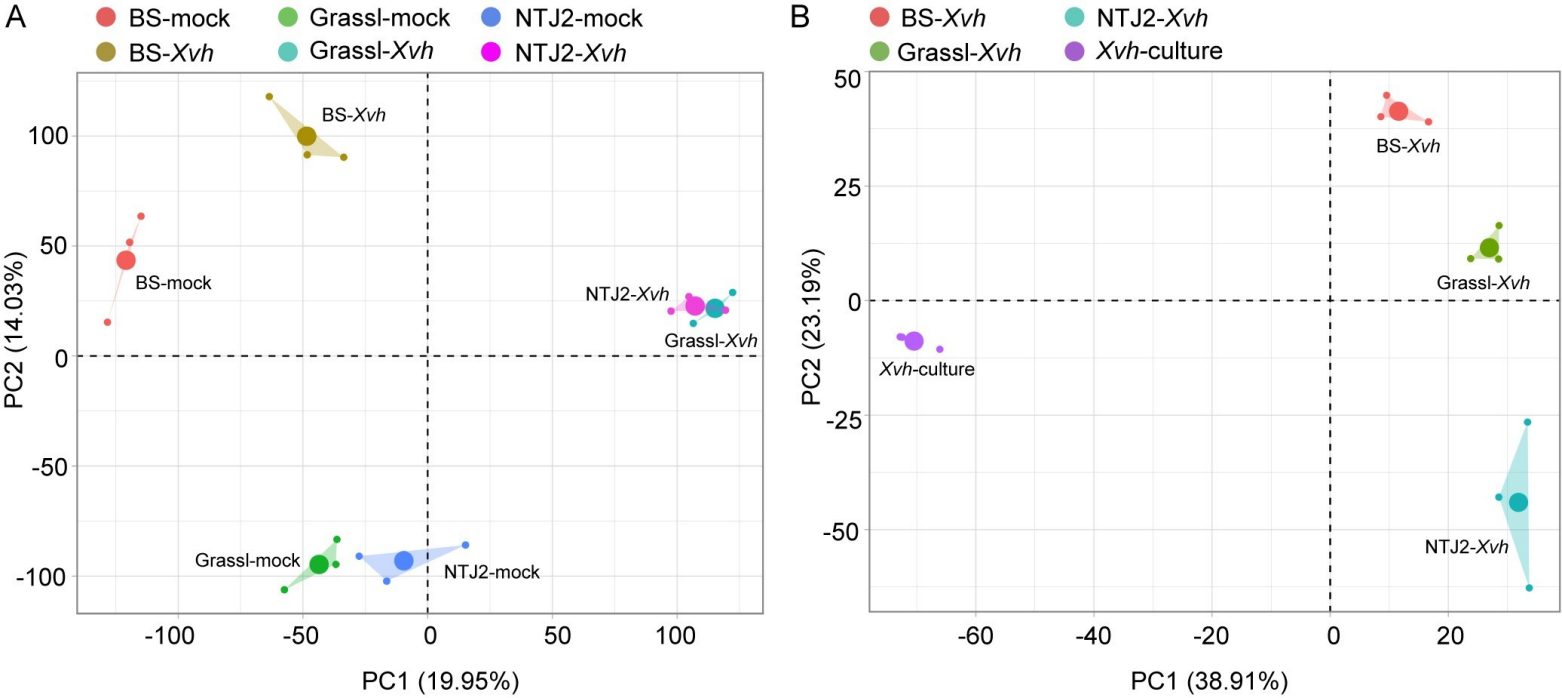
Principal component analysis (PCA) of the dual RNA-seq data. Within each cluster, three small dots represent individual replicates with the larger dot representing the average of the replicates. Reads were mapped against the concatenated reference genome comprised of sorghum nuclear genome, sorghum chloroplast genome, and mitochondrial genome (A) or the *Xvh* genome (B). BS (water-soaked lesions), Grassl (red lesions), and NTJ2 (resistance).

We performed a PCA and confirmed that the biological replicates clustered together (Fig 5). For each sorghum genotype, the transcriptomes of *Xvh* versus mock-inoculated samples were clearly separated (Fig 5A). Within the PCA space, BS-*Xvh* and BS-mock formed a distinct group away from the NTJ2 and Grassl samples. In contrast, NTJ2-*Xvh* clustered with Grassl-*Xvh* and NTJ2-mock clustered with Grassl-mock. This result may reflect relative phylogenetic relatedness between BS, Grassl and NTJ2 and is consistent with the neighbor joining tree described above (S3 Fig). For the *Xvh* transcriptomes (Fig 5B), the *Xvh*-culture vs *in planta* samples showed clear separation along PC1. The three *in planta* samples separated along PC2. Taken together, these data show that the three genotypes of sorghum display distinct transcriptional responses to *Xvh* infection and, further, that the different genotypes of sorghum induce distinct transcriptional responses within *Xvh*.

**Fig 6:**
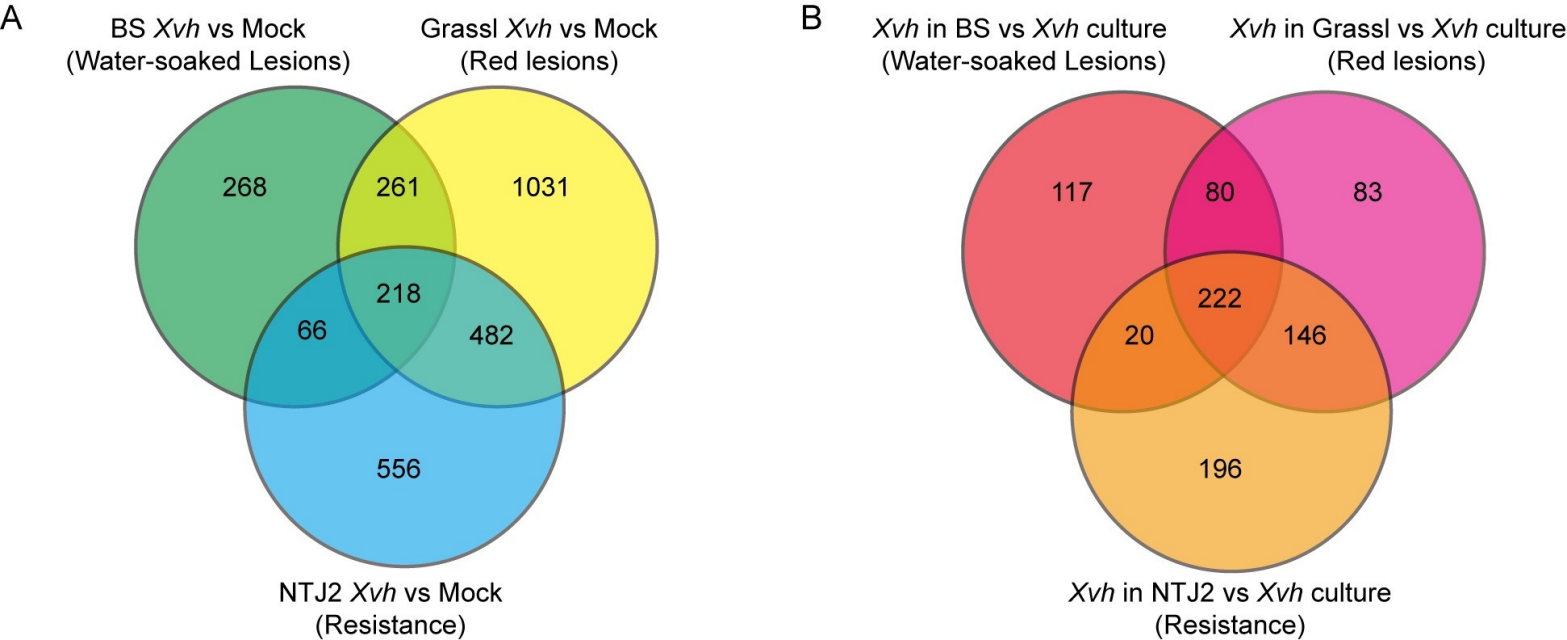
Venn diagram showing the number of differentially expressed genes (FDR adjusted *p* < 0.05; |log_2_ fold change| > 2). A) Sorghum; B), *Xvh*. Complete gene lists are provided in S3 and S4 Tables. BS (water-soaked lesions), Grassl (red lesions), and NTJ2 (resistance).

Next, we identified differentially expressed genes (DEGs) (FDR adjusted *p* < 0.05; abs(log_2_ fold change) > 2) for each genotype of sorghum (*Xvh*-infected vs mock) and compared these gene lists to reveal common and unique transcriptional responses (Fig 6A and S3 Table). In total, comparing *Xvh*-infected and mock-inoculated samples, 813, 1992, and 1322 DEGs were identified from BS, Grassl, and NTJ2, respectively. Among these gene sets, 218 DEGs were common among the three genotypes of sorghum. Consistent with the PCA described above, NTJ2 and Grassl had the most DEGs in common. On the pathogen side, we found that compared to the *Xvh*-culture sample, 439, 531, and 584 *Xvh* genes were up- or down-regulated in BS-*Xvh*, Grassl-*Xvh*, and NTJ2-*Xvh*, respectively (Fig 6B and S4 Table). In addition to 222 DEGs common to all interactions, a number of *Xvh* genes were specifically expressed within each *Xvh*-infected sorghum genotype. Taken together, this analysis identified lists of genes in both the host and pathogen that may explain the phenotypic variation of *Xvh* disease in sorghum.

To further explore these gene expression differences, we performed Gene Ontology (GO) term analysis on the DEGs for each sorghum genotype individually (S5 Fig and S5–S8 Tables). Across all three sorghum genotypes, GO terms associated with protein modification and phosphorylation were the most highly enriched. Surprisingly, defense-related GO terms were not among the top 5 enriched terms for any of the sorghum genotypes, although we note that GO terms associated with defense did occur within the expanded list of enriched terms (S5A Fig and S5 and S6 Tables). Among the *Xvh*-infected transcriptomes tested, only in the NTJ2 background did we observe an enrichment of GO terms for the type III secretion system among the top 5 enriched GO terms. *Xvh* in BS and Grassl shared an enrichment of genes associated with ribosomes (S5B Fig and S7 and S8 Tables).

**Fig 7:**
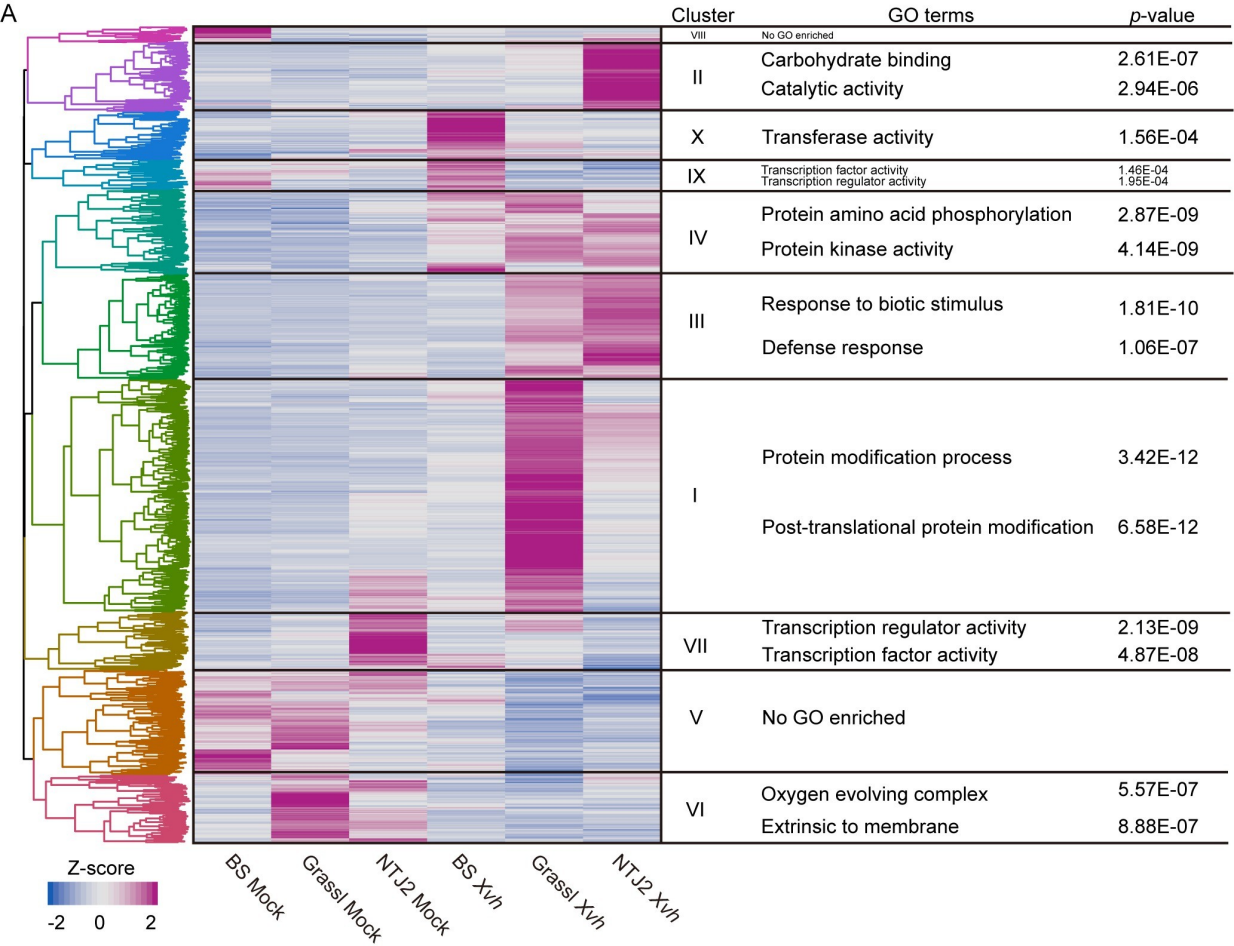
Hierarchical clustering analysis of differentially expressed genes in Sorghum. Analysis based on gene expression from mock and *Xvh*-infected sorghum samples. Sorghum genes identified as differentially expressed in at least one of the pairwise comparisons between mock and *Xvh-*infected sorghum (FDR adjusted *p* value < 0.05, |log_2_ fold change| > 2) were included in this analysis. The transformed Z-score value was generated from FPKM (fragments per kilobase per million mapped sequence reads) values for each condition. The top 2 GO terms based on *p* value (adjusted by the Benjamini-Hochberg method) are listed in this figure. The complete lists of GO terms are provided in S11 Table. BS (water-soaked lesions), Grassl (red lesions), and NTJ2 (resistance).

**Fig 8:**
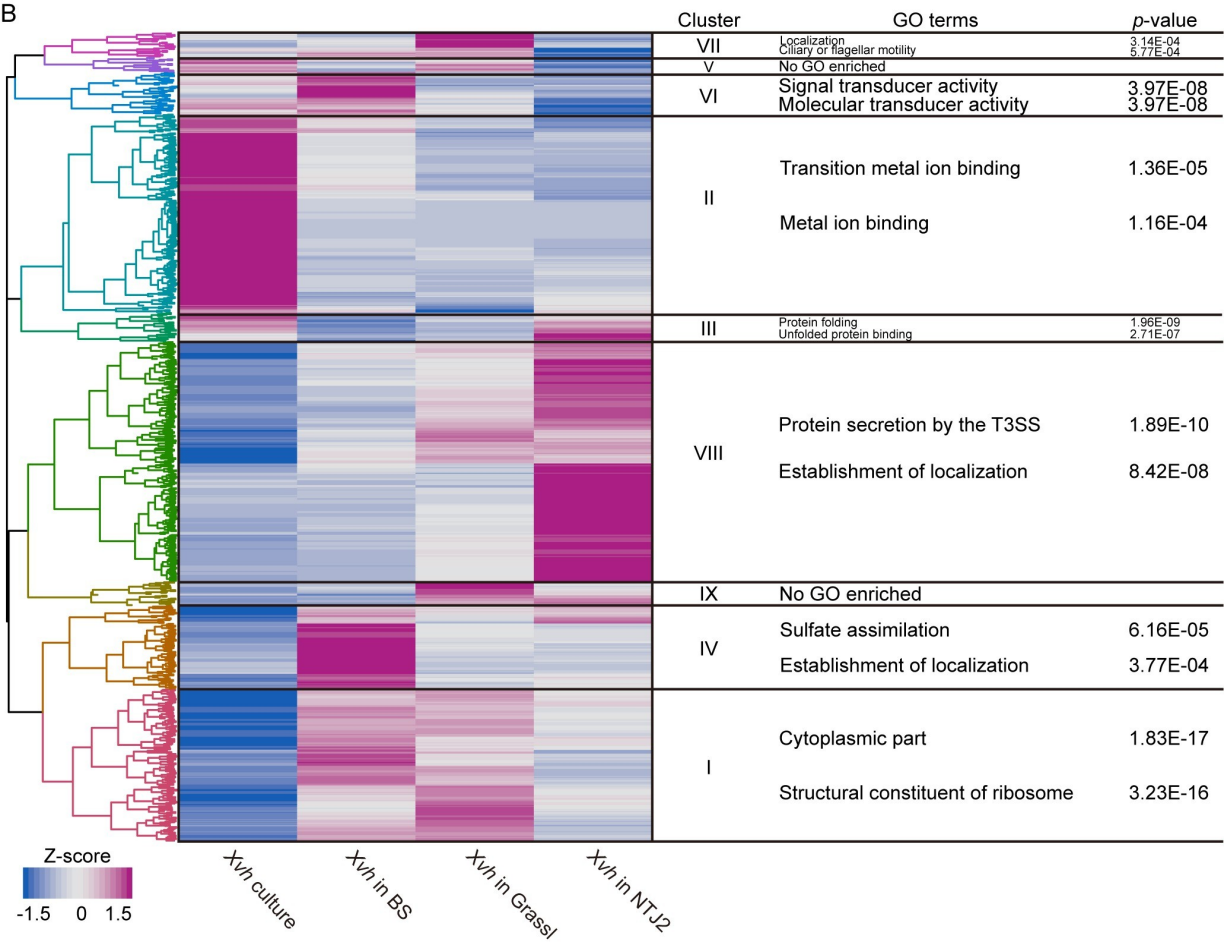
Hierarchical clustering analysis of differentially expressed genes in *Xvh*. Analysis based on gene expression from *Xvh* culture and *in planta* samples. *Xvh* genes identified as differentially expressed in at least one of the pairwise comparisons between *Xvh* culture and *Xvh in planta* (FDR adjusted *p* value < 0.05, |log_2_ fold change| > 2) were used for analysis. The transformed Z-score value was generated from FPKM (fragments per kilobase per million mapped sequence reads) values for each condition. The top 2 GO terms based on *p* value (adjusted by the Benjamini-Hochberg method) are listed in this figure. The complete lists of GO terms are provided in S12 Table. BS (water-soaked lesions), Grassl (red lesions), and NTJ2 (resistance).

We were particularly interested in the gene expression patterns in host and pathogen that result in the water-soaked phenotype. For BS-*Xvh* in Fig 6, we observed 268 and 117 uniquely differentially expressed genes in BS and *Xvh*, respectively. However, GO term enrichment analysis only yielded two terms for *Xvh* (unfolded protein binding and protein folding) and none for sorghum (S9 and S10 Tables). As an alternative approach, we performed hierarchical clustering on z-score transformed gene expression data for all genes that were differentially expressed across at least one comparison (Figs 7 and 8). We compared the results for setting the number of clusters from six to twelve and found that nine and ten clusters separated the gene expression patterns based on the experimental condition for *Xvh* and sorghum, respectively (Figs 7 and 8). Based on this analysis, we found that in clusters IX and X, which show particularly high levels of gene expression in the sorghum BS-*Xvh* interaction, genes associated with transferase and transcription factor activity were overrepresented (Fig 7 and S11 Table). On the pathogen side, we observed a number of genes especially strongly induced in the BS interaction in cluster IV, in which the GO terms ‘sulfate assimilation’ and ‘establishment of localization’ were enriched (Fig 8 and S12 Table). Collectively, these results reveal highly expressed genes in the BS-*Xvh* interaction that may contribute to the water-soaked phenotype.

### Defense- and virulence- gene expression

**Fig 9:**
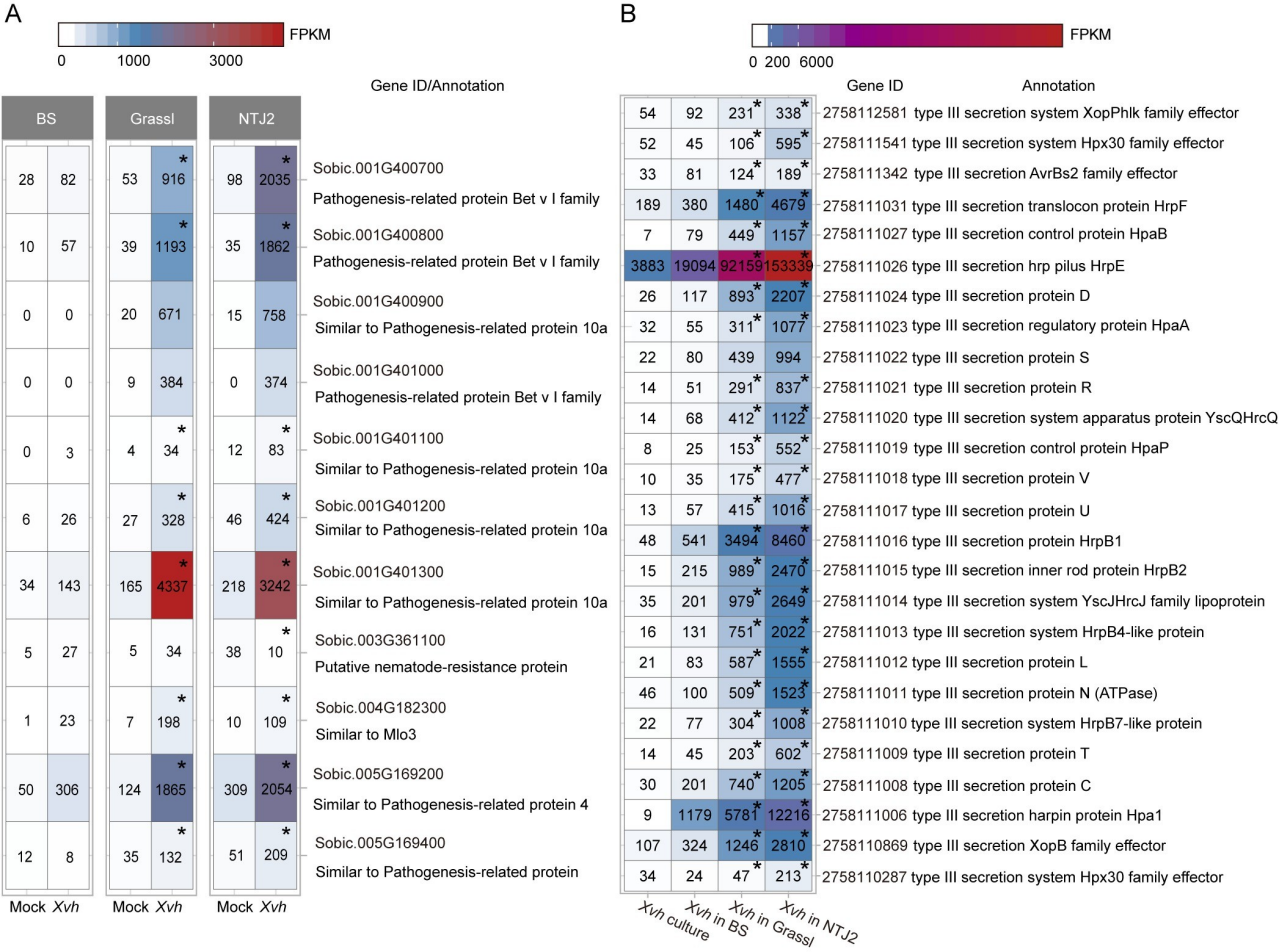
Expression patterns for genes associated with defense and virulence. A) Heatmap showing the expression of defense-related genes in sorghum. These genes were identified based on the associated GO term–defense response. B) Heatmap showing the expression profile of virulence-related genes from *Xvh*. *Xvh* genes related to the T3SS and T3 effectors based on annotation were used in this analysis. The values represent the average of FPKM (fragments per kilobase per million mapped sequence reads) of three replicates for each condition. Asterisks in (A) and (B) indicate significant differential expression (FDR adjusted *p* value < 0.01) as compared to *Xvh* infected BS and *Xvh* in BS, respectively. BS (water-soaked lesions), Grassl (red lesions), and NTJ2 (resistance).

**Fig 10:**
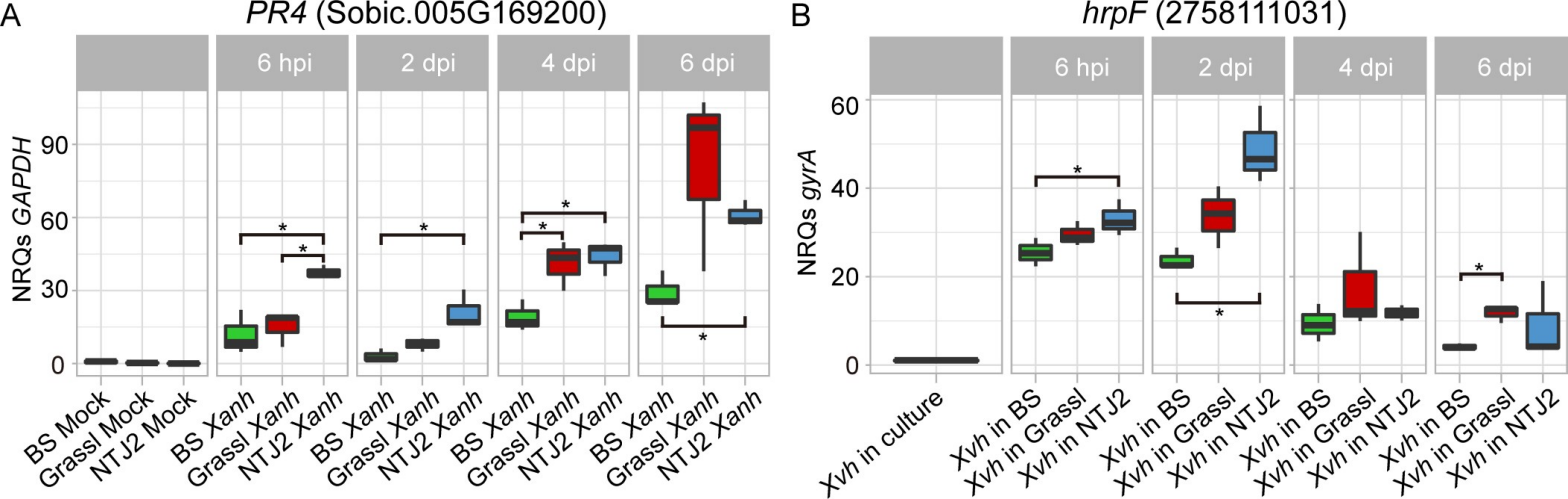
Time course of gene expression of sorghum *PR4* and *Xvh hrpF*. qRT-PCR analysis of sorghum *PR4* (A) and *Xvh hrpF* (B). *PR4* and *hrpF* levels are reported as normalized relative quantities (NRQs) relative to sorghum *GAPDH* and *Xvh gryrA*, respectively. Mean ± s.d.; n = 3 biological replicates. Each plant-mock or plant-*Xvh* replicate contained three inoculation areas from three leaves from three individual plants. *Xvh* inoculum level was OD_600nm_ = 0.5 (~1 × 10^9^ cfu/mL). Each *Xvh* in culture replicate contained ~1 × 10^8^ bacterial cells. Asterisks indicate statistical significance based on unequal variances *t* test (n = 3, *p < 0.05) of pairwise comparisons. BS (water-soaked lesions), Grassl (red lesions), and NTJ2 (resistance).

Based on previous studies from diverse pathosystems, we expected to observe higher expression of sorghum defense-related genes in the resistance interaction compared to the two susceptible interactions. Therefore, we selected a subset of defense-associated genes based on the GO term, “defense response” (S6 Table). As expected, a majority of these genes were more highly expressed in NTJ2-*Xvh* followed by Grassl-*Xvh* and slightly induced in the BS-*Xvh* samples (Fig 9A). On the pathogen side, we hypothesized that genes related to virulence would be most highly expressed in the most susceptible interaction. To test this hypothesis, we investigated the expression of *Xvh* genes related to the T3SS and T3Es genes. Surprisingly, these genes displayed only minor induction during infection of BS and highest induction in the resistant NTJ2 genotype (Fig 9B). These data reveal that, at least among the tested genotypes, defense and virulence-associated genes are most highly expressed during a resistance interaction. We generated a T3SS knockout mutant, *XvhΔhrcC*, by allelic exchange and confirmed that the T3SS is required for full *Xvh* virulence in all three sorghum genotypes (S6 Fig). We hypothesized that the observed gene expression pattern may reflect the specific time point at which the dual RNA-seq experiment was performed. In other words, perhaps T3SS related genes were highly induced either earlier or later in the most susceptible BS interaction. To investigate this hypothesis, we first selected a representative gene from the defense-related- (*PR4*) and virulence-related- (*hrpF*) gene lists from sorghum and *Xvh*, respectively, and assessed relative expression by qRT-PCR in the samples that were used for RNA-seq (S7 Fig). We confirmed similar expression profiles from both qRT-PCR and RNA-Seq for the selected genes. Next, we quantified the expression levels of each gene across four time points (6 hours, 2 days, 4 days, and 6 days post-inoculation) in independent biological samples (Fig 10). Consistent with the RNA-seq data, the expression of *PR4* was induced most strongly in the resistance interaction, especially at early time points. By 4 dpi and 6 dpi, the expression of *PR4* in Grassl and NTJ2 was similar and higher than in BS (Fig 10A). The T3SS gene *hrpF* was most strongly upregulated at the early time points [6 hours post-inoculation (hpi) and 2 days post-inoculation (dpi)] and, consistent with the RNA-seq data, this gene was most highly upregulated in the resistance interaction. At 4 and 6 dpi, expression of *hrpF* was lower in all sorghum genotypes (Fig 10B). In summary, the qRT-PCR analysis confirms and supports what was observed using transcriptomics, the expression of *hrpF* and *PR4* is highest in the resistance interaction. Together, our data indicates that plant defense- and pathogen virulence-related genes are most highly induced during a resistance interaction in the sorghum-*Xvh* pathosystem.

### Considering pathogen virulence responses within a distinct pathosystem

**Fig 11:**
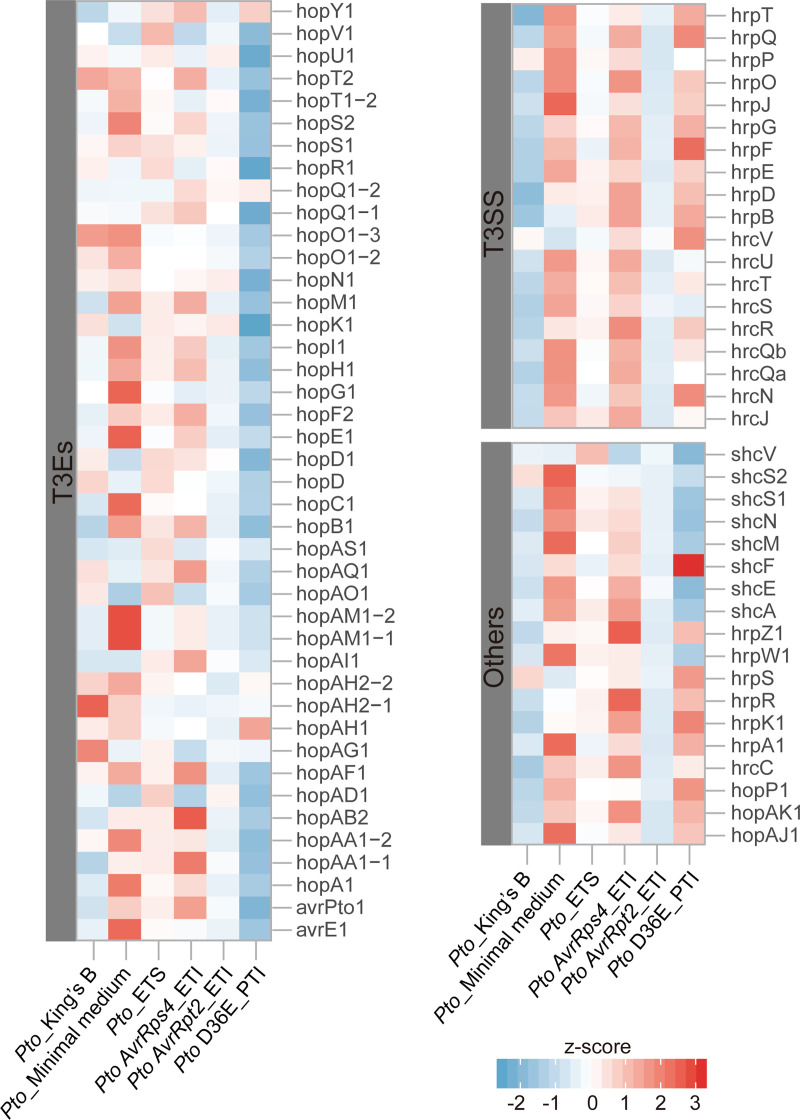
Expression profile of the T3SS and T3E genes of *Pseudomonas syringae* pv. *tomato* DC3000 (*Pto*) in T3SS/T3E-inducible minimal medium versus nutrient-rich King’s B and across a range of *Arabidopsis thaliana* lines with different ETS, ETI and PTI phenotypes. *Pto*, *Pto* AvrRps4 (*Pto* strain ectopically expressing T3E AvrRps4), *Pto* AvrRpt2 (*Pto* strain ectopically expressing T3E AvrRpt2), and *Pto* D36E (*Pto* mutant strain lacking all 36 known *Pto* T3Es) infection in *Arabidopsis thaliana* Col-0 with ETS, ETI, ETI, and PTI phenotypes, respectively. T3SS, type III secretion system. T3E, type III effectors. Others, T3SS helper and chaperone. The transformed Z-score value was generated from FPKM (fragments per kilobase per million mapped sequence reads) values for each condition. The raw sequencing data was generated by Nobori et al. 2018 [25].

Our results indicate that both sorghum defense-related genes and *Xanthomonas* virulence-related genes are most highly up-regulated in NTJ2, a resistance interaction. To understand whether this pattern was specific to NTJ2 sorghum and *Xvh* or more broadly representative of host-pathogen interactions, we considered a previous study on the bacterial pathogen *P*. *syringae* pv. *tomato* DC3000 (*Pto*) and its plant host, *Arabidopsis thaliana*. In this previous study, the authors characterized pathogen gene expression when grown on minimal media versus nutrient-rich King’s B and during infection of wildtype *Arabidopsis* and several mutant lines with different PTI, ETS, and ETI phenotypes [25]. Because our RNAseq data analysis pipeline differed slightly from that used by Nobori et al. [25], we choose to re-analyze the dataset using our methods for consistency. Within the *Pto* transcriptome data, many genes related to the T3SS and T3Es were induced in *Pto* during both PTI and ETI interactions (PTI: *Pto* D36E (*Pto* mutant strain lacking all 36 known *Pto* T3Es); ETI: *Pto* AvrRps4 (*Pto* strain ectopically expressing T3E AvrRps4)). In contrast, *Pto*-triggered ETS in wildtype *A*. *thaliana* and ETI triggered by AvrRpt2 both showed less induction of T3SS and T3E genes in terms of both the number of genes and magnitude of differential expression (Figs 11 and S8). Taken together, these findings reveal multiple different snapshots of the continually evolving host-pathogen arms race.

### Impact of nutrients on *Xvh* virulence response

**Fig 12:**
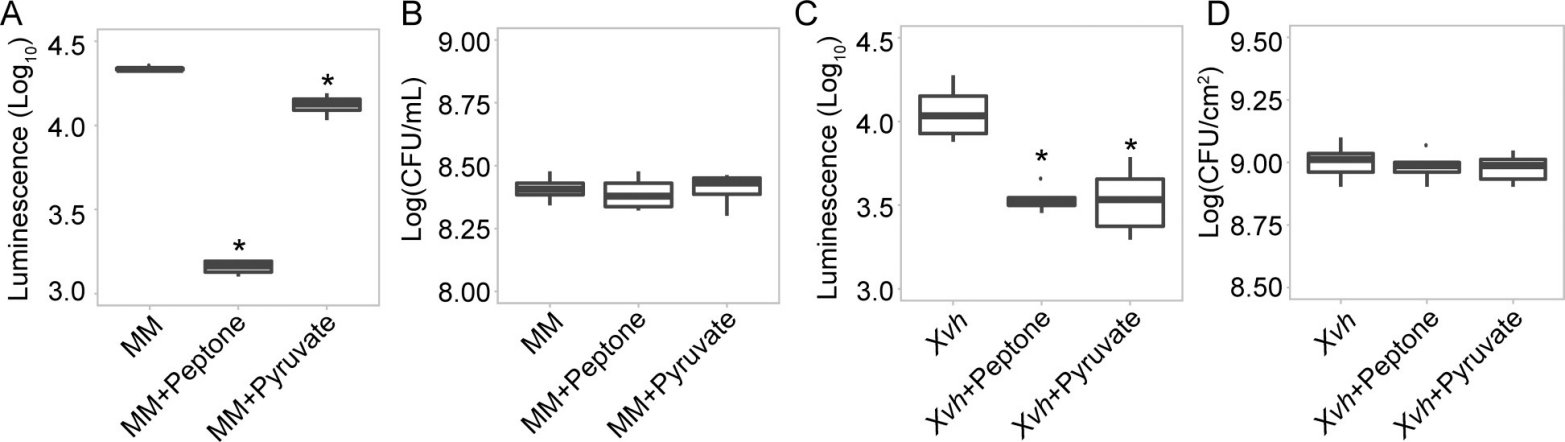
Impacts of nutrients on the expression of T3SS-associated genes of *Xvh in vitro* and *in planta*. (A-B) *Xvh* expressing *hrpF* fused to a luciferase (Luc) reporter [*Xvh* (*hrpF*_promoter_*Luc*)] grows in T3SS/T3E-inducible minimal media (MM) in absence or presence of peptone (2%) and pyruvate (20 mM), respectively. Bacteria were cultured for 12 hours and then adjusted to OD_600nm_ = 0.6 for assaying luciferase activity (A). (B) Bacterial populations (OD_600nm_ = 0.6) were determined and are shown as colony-forming units (CFU). Mean ± s.d.; n = 4 biological replicates. Asterisks indicate statistical significance based on unequal variances *t* test (n = 4, *p < 0.05) comparison with MM treatment. (C-D) Sorghum NTJ2 leaves were infected with *Xvh* (*hrpF*_promoter_*Luc*) (OD_600nm_ = 0.5 (~1 × 10^9^ cfu/mL)) in the absence or presence of the indicated nutrients. Luciferase activity assay was performed at 48 hpi (C). Peptone and pyruvate were used at 0.5% (w/v) and 20 mM, respectively. (D) Bacterial populations were determined at 48 hpi and are shown as colony-forming units (CFU). Mean ± s.d.; n = 5 biological replicates. Asterisks indicate statistical significance based on unequal variances *t* test (n = 5, *p < 0.05) comparison with *Xvh* infection.

The above results suggest two possibilities: 1) The *in planta* environment encountered by *Xvh* upon colonization of different sorghum genotypes leads to variable virulence responses; 2) Colonization by *Xvh* of different sorghum genotypes triggers different host responses that in turn lead to variable virulence responses. In other words, are the observed differences in virulence response a reflection of *a priori* differences between the tested sorghum genotypes, or alternatively, differences in how the sorghum genotypes respond to colonization by *Xvh*? To investigate these two possibilities, we treated *Xvh* expressing the T3SS gene *hrpF* fused to a luciferase (Luc) reporter [*Xvh* (*hrpF*_promoter_*Luc*)] with apoplastic fluid collected from the different sorghum genotypes, resistant (NTJ2) and susceptible (BS). Growth of *Xvh* in minimal medium (MM) induces expression of *hrpF_*promoter*_Luc*. Twelve hours after treatment with apoplastic fluid, we observed the expression of the *hrpF-luc* reporter at similar levels in both tested genotypes (S9 Fig). This time point was chosen based on previously published experiments [26] and the result suggests that the apoplastic environment is similar between the resistant and susceptible sorghum genotypes, prior to inoculation, with respect to virulence gene expression. Our GO term analysis on the *Xvh* DEGs for each sorghum-*Xvh* interaction showed that the GO terms associated with carbon/nitrogen source biosynthetic and metabolic processes including cellular nitrogen compound biosynthetic process, organic acid metabolic process, and tricarboxylic acid (TCA) cycle were specifically enriched in BS, but not in NTJ2 and Grassl background (S8 Table). In addition, previous work from *Xanthomonas* and *Pseudomonas* pathosystems demonstrated that nutritional signals can affect the virulence response of bacterial pathogens [26–30]. Therefore, we hypothesized that during infection, nutrients within the apoplastic environment might be differentially changed among the sorghum genotypes, which in turn influences the bacterial virulence response. To determine whether nutrients are able to impact the virulence response of *Xvh*, we measured the activity of *hrpF* in *Xvh* (*hrpF*_promoter_*Luc*) growing in minimal media containing peptone (carbon and nitrogen source) and pyruvate (carbon source), individually. Peptone and to a lesser degree, pyruvate, were able to repress the induction of *hrpF* (Fig 12). Further, when either peptone or pyruvate was co-infiltrated into NTJ2 sorghum leaves with *Xvh*, the induction of *hrpF* was significantly reduced (Fig 12). These data are consistent with previous studies in *Pseudomonas syringae* and *Xanthomonas campestris* pv *vesicatoria* [26,28]. These data support the conclusion that the differential expression of T3SS genes is the result of differences in the nutrients/metabolites that accumulate *in planta* after inoculation with the pathogen. Peptone is a complex mixture of polypeptides and amino acids. Our GO term analysis on the *Xvh* DEGs showed that genes related to the sulfur amino acid biosynthetic and metabolic processes were overrepresented in BS-*Xvh* interaction (water-soaked lesions phenotype) (S8 Table). We therefore tested whether sulfur-containing amino acids (methionine and cysteine) could repress *Xvh* virulence gene expression (S10 Fig). There was no observable effect of these treatments (methionine and cysteine) on the expression of *hrpF in vitro* and *in planta* (S10 Fig). This indicates that the impact of peptone on the *Xvh* virulence response may be attributed to an amino acid mixture, other nutrients or alterations in the environment and future studies will investigate this further.

## Discussion

*Xanthomonas* is an important genus of gram-negative bacteria that causes severe disease on hundreds of plant hosts, including economically important crops [8]. *Xvh* causes bacterial leaf streak of sorghum and was identified several decades ago [31]. However, the molecular basis underlying the arms race between sorghum and *Xvh* is still unclear. In this study, we conducted a large-scale screen for resistance or susceptibility to *Xvh* in 156 diverse sorghum genotypes covering five basic and ten intermediate races in sorghum and observed three general phenotypes: water-soaked lesions, red lesions, and resistance. While each sorghum variety showed a primary phenotype, we note that some varieties showed a minor secondary phenotype. For example, several varieties primarily developed a classic ‘water-soaked’ lesion but also showed a few red spots (S1 Fig). By using dual RNA-seq analysis, we were able to study transcriptome profiles of both the sorghum plant and *Xvh* bacteria, simultaneously. We identified a large number of differentially expressed genes that are commonly and specifically induced or suppressed across the three *Xvh* disease phenotypes (Fig 6). Of particular interest, we observed that the expression of some bacterial T3SS/T3Es and plant defense-related genes were strongly induced in the resistance interaction. In contrast, these genes were only weakly induced in the most susceptible host (Fig 9). This unexpected result was further confirmed using time-course expression analysis (Fig 10). Reanalysis of a previously published dataset from *Arabidopsis* infected with *Pseudomonas syringae* also showed a similar pattern of heightened virulence in the context of ETI triggered by AvrRps4 (Figs 11 and S8). Moreover, we found that specific metabolites (e.g., peptone and pyruvate) were able to repress the virulence response *in vitro* and *in planta* (Fig 12). Together, these results provide a unique insight into a bacterial disease of sorghum and shed light on our understanding of the molecular arms race between host and pathogen.

High-throughput transcriptome sequencing (RNA-seq) is an economical and powerful tool for studying transcriptional responses in diverse organisms [32]. To date, there have been very few studies simultaneously examining transcriptional changes in plant bacterial pathogens and their hosts, primarily due to the technical challenges associated with these experiments. For example, compared to the plant transcriptome, the *in planta* bacterial transcriptome is underrepresented leading to lower coverage of bacterial sequences. In addition, efficient rRNA depletion in total mixed RNA can be challenging [22,33]. Recently, a special RNA-seq approach, referred to as “dual RNA-seq,” was developed to simultaneously capture microbe and host transcriptional profiles [22]. In our study, we overcame the technical challenges associated with dual RNA-seq by inoculating the sorghum leaves with a high concentration of bacteria (OD_600nm_ of 0.5, ~10^9^ cfu/mL) and performing rRNA depletion of total mixed RNA from both plants and bacteria to enrich for mRNA. Based on the PCA results, biological replicates clustered together and the variation across diverse samples was evident (Fig 5). The gene expression values obtained from RNA-seq data were validated by qRT-PCR using the same RNA samples for RNA-seq and new independent RNA samples, indicating the reliability of our RNA-seq data (Figs 10 and S7).

In this study, we were particularly interested in the water-soaked lesion phenotype observed in BS. These symptoms are typical of diverse Xanthomonads including *X*. *citri* pv. *citri*, *X*. *citri* pv. *malvacearum*, *X*. *axonopodis* pv. *manihotis*, and *X*. *oryzae* pv. *oryzae* [20,24,34,35]. In many cases, the water-soaked lesion has been linked to expression of a specific class of T3Es, transcriptional activator-like (TAL) effectors, that induce expression of plant sugar transporters [36]. However, the *Xvh* genome does not encode TAL effectors [16]. Through hierarchical clustering, we identified upregulated *Xvh* genes associated with the GO term ‘sulfate assimilation’ in BS (Fig 8). Several observations have established a connection between sulfur metabolism and virulence traits of various bacterial pathogens. For example, MsrA, a methionine sulfoxide reductase, is required for full virulence of the plant pathogen *Erwinia chrysanthemi* [37]; and CymR, the master regulator of cysteine metabolism in *Staphylococcus aureus*, plays an important role in adaptation and survival in the host [38]. On the host side, the GO term ‘transferase activity’ was associated with *Xvh*-infected BS (Fig 7). Because ‘transferase activity’ is vague, this GO term may be less informative. Still, we note that a number of plant genes with the function of “transferase activity”, like *glycosyltransferase-like RSE1* from *Arabidopsis thaliana* have been reported as a negative regulator of defense response [39,40]. Therefore, these genes identified in *Xvh*-BS interaction can be considered as possible candidates for the water-soaked lesion phenotype and will be investigated in future studies.

In response to attack by pathogens, plants have developed sophisticated immune responses such as PTI and ETI. In turn, pathogens have evolved a wide range of strategies to interfere with the plant immune system. This dynamic and complex molecular process was described as the four stage zig-zag model by Jones and Dangl in 2006 [1]. In this model, a stronger plant defense response equates with increased resistance to a pathogen (S11 Fig). This model does not, however, incorporate the strength of virulence on the pathogen side. Our transcriptome analysis revealed that the expression of many plant defense-related and pathogen virulence-related genes was most strongly induced in the resistance interaction (Figs 9 and 10). This unexpected result was further confirmed in a time-course expression analysis and reanalysis of a previously published dataset from *Arabidopsis* infected with *Pseudomonas syringae* [25]. In addition, a similar interaction pattern was previously observed in the rice-*X*. *oryzae pv*. *oryzae* pathosystem and *Staphylococcus aureus* infection model in mice [41,42]. Here, we equate a number of genes and magnitude of expression with the strength of the response while also acknowledging several caveats. mRNA quantity does not always correlate with protein levels [43]. A proteomics approach may be pursued though, at present, that type of analysis remains technically challenging. It is also possible that the proteins accumulate within the bacteria but are not secreted into plant cells, however, this seems unlikely. Further, not all defense and virulence-related genes contribute equally to the response, and we are also looking at just one stage of the pathogen life cycle. Despite these caveats, we propose a new dimension, the strength of the pathogen virulence response, to the zig-zag model of host-pathogen interactions and suggest that in some cases, the plant resistance response is a form of escalation that correlates with an increased virulence response from the pathogen (S11 Fig).

Our findings raise the following question: Why is the expression of the T3SS and T3Es stronger in resistant hosts than susceptible hosts during infection? One possibility is that nutrients in the apoplast might be different between resistant and susceptible sorghum genotypes before infection. For example, *Xvh* cells may encounter a nutrient-poor environment in resistant genotypes and therefore induce virulence genes in an attempt to induce the production of specific metabolites; a resistance interaction prevents the bacteria from altering this environment and so virulence gene expression remains high. In contrast, a susceptible sorghum genotype may have a nutrient-rich apoplast environment that both allows the bacteria to proliferate and thus triggers a reduction in expression of virulence genes. To investigate this hypothesis, we treated *Xvh* cells with apoplastic fluid from the different sorghum genotypes. However, we did not observe a difference in virulence gene induction (S9 Fig) and therefore conclude that it is unlikely that pre-infection differences between the sorghum genotypes explain the observed differences in virulence gene induction. In addition, *in vitro* and *in planta* assays showed that a specific metabolite, pyruvate, was able to repress the induction of the T3SS (Fig 12). We followed an established protocol [25] but cannot rule out some amount of cytoplasmic contamination during apoplastic fluid extraction. Nevertheless, even if some cytoplasmic fluid was released, this would have been similar between the genotypes and may also accurately reflect the conditions experienced by the bacteria *in planta*. Together, these data suggest that upon infection of the susceptible BS sorghum genotype, *Xvh* successfully induces the plant host to produce specific metabolites that subsequently repress virulence gene expression, perhaps as a mechanism to avoid further defense response induction. If there is a fitness trade-off between bacterial virulence and elicitation of plant immunity, *Xvh* would tightly regulate the expression of genes related to T3SS and T3Es during infection of susceptible hosts to minimize elicitation of plant defenses. In contrast, in the context of resistance, host defenses may trigger increased expression of T3SS and T3E genes which in turn trigger stronger plant defense responses and escalation of the host-pathogen arms race.

## Materials and methods

### Plant material and growth conditions

156 sorghum genotypes were evaluated for their response to strain *X*. *vasicola* pv. *holcicola* BLS185 inoculation (S1 Table). Qualitative disease phenotypes (water soaking lesions, red lesions, resistance) were assigned for each variety based on the primary symptom type (S1 Fig). Among these genotypes, Black Spanish (BS), Grassl, and NTJ2 were used in this study for Dual RNA-seq. The plants were grown in a growth chamber set at 28/23°C with 14/10 h (day/night) conditions and 50% relative humidity. 14-day-old plants were used for all experiments.

### Bacteria strain preparation and bacterial mutant generation

*X*. *vasicola* pv. *holcicola* BLS185 (*Xvh*) was used in this study (https://img.jgi.doe.gov/cgi-bin/m/main.cgi?section=TaxonDetail&page=taxonDetail&taxon_oid=2757320517). For generating the *XvhΔhrcC* mutant, an allelic exchange strategy [44] was employed with some modifications. In brief, two sequence fragments for the upstream and downstream homologous regions of the target gene were cloned into the sucrose counter-selection allelic exchange vector pDEST2T18ms by Gateway technology (pDEST2T18ms was a gift from Brian Kvitko (Addgene plasmid # 72647; http://n2t.net/addgene:72647; RRID:Addgene_72647)). For generating *Xvh* (*hrpF*_promoter_*Luc*), two sequence fragments for the promoter region of *Xvh hrpF* and coding region of *luciferase* (*Luc*) from vector pGWB35 were cloned into the vector pENTR/D-TOPO. The sequence fragment of *hrpF*_promoter_*Luc* was amplified by PCR and then cloned into EcoRI digested vector pVSP61des by In-Fusion HD Cloning Plus cloning system. The recombinant plasmids were verified by Sanger sequencing and subsequently transferred into the wild-type *Xvh* strain via electroporation. Briefly, 2 μg plasmid was added to 100 μl of electrocompetent cells and mixed; mixtures were placed in a pre-chilled, sterile, 2mm electroporation cuvette. The cells were electroporated with a BioRad Gene Pluser II electroporation system (Bio-Rad Laboratories) at 2.5 kV, 25 μF, and 50 Ω and were then added to a fresh 1 ml of NYG media and incubated at 30°C for 3h. Cells were then plated on NYGA supplemented with appropriate antibiotic. Resistance clones were selected. For generating the *XvhΔhrcC* mutant, Sucrose^R^ mutants were further screened in NYGA medium containing 10% sucrose with appropriate antibiotic. The mutants were further identified on selective media and mutations were verified by PCR product sequencing. The primers are listed in S13 Table.

### Bacterial infection and sampling

*Xvh* strain was grown on plates containing NYGA medium (per liter: 5.0g peptone, 3.0g yeast extract, 20.0g glycerol, and 1% agar) for 48 hours at 30°C. Next, bacteria were harvested from the plates as “*Xvh*-culture” samples for RNA-seq. For the inoculation experiments, the harvested bacteria were suspended in sterile 10mM MgCl_2_ to an OD_600nm_ of 0.5 (~10^9^ cfu/mL, for inoculation of the Dual RNA-seq samples and qRT-PCR samples) or 0.02 (~10^7^ cfu/mL for other inoculation experiments). The fourth leaf of 14-day-old sorghum plants was syringe-inoculated with bacterial suspensions using a needleless syringe. The leaves were inoculated with 10mM MgCl_2_ as the mock treatment. The inoculation time of the day was 1–3 pm. The infected tissue areas (~2cm × 0.5cm) in leaves were harvested at 0 hours post-inoculation (hpi), 6 hpi, 2 days post-inoculation (dpi), 4 dpi, and 6dpi. All the bacterial and plant samples were immediately frozen in liquid nitrogen after harvest and stored at −80°C. All experiments were performed with three biological replicates unless otherwise indicated.

### Bacterial growth assay

At each time point, one sorghum leaf disc (3 mm diameter) was cut around the inoculation point using a cork-borer. Two leaf disc sections from two inoculation areas in one leaf on one plant were combined per replicate. Replicate sizes are indicated in each figure legend. Samples were ground using Qiagen Tissuelyser (2 minutes, at 30 Hz) in 10 mM MgCl_2_ with a 3-mm glass bead in a 2.0 mL Eppendorf Safe-Lock tube. Serial dilutions were plated on NYGA medium with appropriate selection plus cycloheximide to inhibit fungal growth. Log_10_-transformed colony-forming units (cfu) per cm^2^ leaf surface area were calculated to estimate bacterial populations.

### *In vitro* treatments of bacteria with apoplastic fluid and nutrients

Extraction of apoplastic fluid was performed following previous publications with some modifications [25,27]. The fourth leaf of 14-day-old sorghum plants was syringe-inoculated with sterile Milli-Q water (Millipore, Inc.). The infected tissue areas in leaves (1 gram) were harvested at 48 hours post-inoculation. The tissues were cut into segments of 2 cm, placed in a 60-mL syringe with 10 mL cold sterile Milli-Q water and vacuum-infiltrated by pulling the plunger. Infiltrated leaves were darker than the non-infiltrated and quickly sank. Leaf segments were incubated for 2 hours. The samples were passed through a cell strainer (FALCON, #352340) in 50-mL polypropylene conical tubes by centrifugation at 400 *g* for 5 min. The obtained fluid, now referred to as apoplastic fluid, filtered (0.22 μM), was stored at −70°C until needed. For *in vitro* treatments of bacteria with apoplastic fluid and nutrients, *Xvh* (*hrpF*_promoter_*Luc*) was grown to mid-log phase in NYG medium at 30°C, washed twice in 10 mM MgSO_4_, and resuspended to an optical density of OD_600nm_ (OD_600nm_ = 0.6 ~ 0.8) in minimal media (MM). For *in vitro* treatments of bacteria with apoplastic fluid, the bacterial suspension was mixed with apoplastic fluid (total volume = 2 mL; mixing ratio = 5:1; start OD_600nm_ = 0.6), or sterile Milli-Q water for the control. For *in vitro* treatments of bacteria with nutrients, bacterial suspension was mixed with peptone (2%), cysteine (0.06%), methionine (0.06%), and pyruvate (20 mM), respectively (start OD_600nm_ = 0.6).

Bacteria were cultured for 12 hours and 2 mL of bacterial suspension was harvested by centrifugation at 13,000 *g*, 2 min. The supernatant was discarded and the pellet was resuspended with 2 mL sterile Milli-Q water. After adjustment to an OD_600nm_ = 0.6, 1 mL of the bacterial suspension was added to 1.5 mL Eppendorf tube. Bacterial cells were harvested by centrifuge at 13,000 *g*, 2 min. The supernatant was discarded and the pellet was resuspended with 100 μL sterile Milli-Q water for luciferase activity assay.

### Luciferase activity assay

For each bacterial sample, 50 μl of bacterial suspension was placed in a black 96-well plate. For each leaf sample, two sorghum leaf discs (3 mm diameter) were cut around the inoculation point using a cork-borer, and samples were placed in a black 96-well plate containing 50 μl sterile Milli-Q water (Millipore, Inc.). 50 μL luciferin solution [10 mM luciferin (D-Luciferin, Potassium Salt, GOLDBIO #LUCK-100) and 1% Triton X-100 in sterile Milli-Q water] was pipetted into each sample. Luminescence was measured at room temperature by plate reader Infinite 200 within 20 seconds.

### RNA extraction

Total RNA was extracted using the TRIzol Reagent (Invitrogen). Each plant-mock or plant-bacteria RNA sample contained three inoculation areas from three leaves from three individual plants. Each bacterial RNA sample contained ~1 × 10^8^ cells. The aqueous phase containing the RNA was mixed with an equal volume of 70% ethanol, and then applied to a column from the Spectrum Plant Total RNA Kit (Sigma-Aldrich) following the manufacturer’s instructions, including the On-Column DNase Digestion protocol. RNA quality was checked using the Agilent 2100 Bioanalyzer (Agilent Technologies).

### rRNA depletion, cDNA library generation, and sequencing

Ribosomal RNA of sorghum-mock samples and *Xvh* in culture samples was removed using the Ribo-Zero rRNA Removal Kit (Plant Leaf) (Illumina) and the Ribo-Zero rRNA Removal Kit (Bacteria) (Illumina), respectively. 2 μg DNase-treatment total RNA was used for rRNA depletion. For the sorghum- *Xvh* mix samples, 8 μl rRNA Removal Solution (Plant Leaf), and 8 μl Ribo-Zero Removal Solution (Bacteria) were mixed for use in rRNA depletion. The protocol, as stated in the user guide, was implemented. cDNA libraries for Illumina sequencing were generated with the NEBNext Ultra II Directional RNA Library Prep Kit for Illumina by Novogene Corporation, California. HiSeq platforms with paired-end 150 bp (PE 150) sequencing strategies were used for sequencing by Novogene. The RNA-sequencing data has been deposited in the National Center for Biotechnology Information Gene Expression Omnibus database (accession no. GSE142035).

### Reads mapping and differential gene expression analysis

Reads were trimmed with Trimmomatic [45]. HISAT2 (version 2.0.6) [46,47] was used to align the RNA-seq reads against a concatenated genome comprised of the sorghum nuclear genome (version Sorghum bicolor v3.1.1; https://phytozome.jgi.doe.gov) [48], the sorghum chloroplast genome (GenBank: EF115542.1) [49], the sorghum mitochondrial genome (NCBI Reference Sequence: NC_008360.1), and the *Xvh* genome to allow for the best alignment of each read. Default parameters were used except for the inclusion of the '—dta-cufflinks' parameter. The resulting SAM (Sequence Alignment/Map) files (.sam) were sorted and converted to BAM (Binary Alignment/Map) files (.bam) using SAMtools [50]. Stringtie (version 1.3.5) [47] was used to perform quantification, generating FPKM (Fragments Per Kilobase of transcript per Million mapped reads) values. The parameter -e was used to limit quantification to alignments matching the reference annotation file. This was performed twice, once to quantify the reads mapped to the sorghum genome and again to quantify the reads mapped to the *Xvh* genome. Differential expression analysis was carried out separately for the sorghum and *Xvh* using Cuffdiff [51] with non-default parameters "library-normalization-method classic-fpkm," "dispersion-method per-condition," and "max-bundle-frags 500000000".

Library normalization was done as described above so that the quantification from Stringtie would exactly match the differential expression mean estimations from Cuffdiff. Similarly, this dispersion method was used so that each mean estimation would get its own variance estimation, and an unequal variance test could be performed for differential expression.

PCA was performed in the R environment using the Ballgown and FactoMineR packages [52,53] and the Ballgown FPKM values were calculated by Stringtie with the -b parameter.

Hierarchical clustering analysis was done in the R environment. For both the *Xvh* and sorghum host transcriptomic heatmaps, only genes that were significantly differentially expressed (FDR adjusted *p* < 0.05; abs(log_2_ fold change) > 2) in at least one comparison were used in the dendrogram formation. Genes were then center-scaled by subtracting the mean and dividing by the variance, hereafter referred to as z-transformed, to normalize for inherent expression differences between genes. The dendrogram was created using base R functions, dist and hclust from the stats package (v3.4.4), which uses Euclidean distance and complete agglomeration respectively. Edge and node colors were assigned using a heuristic approach in dendextend R package (v1.6.0). The z-transformed FPKM for each condition is shown with the color gradient.

Enriched GO terms were identified using the BiNGO plugin for Cytoscape [54]. The Hypergeometric test with the Benjamin and Hochberg false discovery rate (FDR) multiple testing correction was applied at a significance level of ≤0.05.

### qRT-PCR Analysis

qPCR assays were performed on a CFX384 Touch Real-Time PCR Detection System (Bio-Rad) using the SYBR Select Master Mix (Thermo Fisher). 1 μg DNase-treatment RNA was converted to cDNA and used for qRT-PCR. Normalized relative quantities (NRQs) were calculated as described by Hellemans et al. (2007) [55]. The *gyrA* gene (ID: 2758113003) of *Xvh* and the *GAPDH* gene (Sobic.010G262500) of sorghum were used as the endogenous controls for gene expression analysis. For each biological sample, three technical replicates were performed. Normalized relative quantities of *Xvh* and sorghum genes for all replicates were further normalized as a ratio to the geometric mean of *Xvh* in culture samples and sorghum BS mock samples (6 hpi, inoculation with 10 mM MgCl_2_), respectively. The primers specific for genes of interest are listed in S13 Table.

### Reanalysis of the *Pto* transcriptome data from Nobori et al. 2018

The *Pto* transcriptome data from Nobori et al. 2018 [25] was reanalyzed to identify the expression pattern of virulence genes *in planta*. The transcriptome data of a set of samples (*Pto* on King’s B medium; *Pto* on minimal medium; *Pto*, *Pto* AvrRps4, *Pto* AvrRpt2, and *Pto* D36E infection in *Arabidopsis thaliana* Col-0 with ETS, ETI, ETI, and PTI phenotypes, respectively) were obtained from the National Center for Biotechnology Information Gene Expression Omnibus database (accession no. GSE103442). The RNA-seq reads were mapped onto the *Pto* DC3000 genome/coding sequence (CDS) (Pseudomonas Genome Database: https://www.pseudomonas.com/) using HISAT2 (version 2.0.6) [46,47]. The resulting SAM (Sequence Alignment/Map) files (.sam) were sorted and converted to BAM (Binary Alignment/Map) files (.bam) using SAMtools [50]. Stringtie (version 1.3.5) [47] was used to perform quantification, generating the Ballgown FPKM values. The genes with type III annotations were selected and z-transformed, to normalize for inherent expression differences between genes.

### Sorghum phylogenetic tree and PCA analysis

GBS-based SNP data for the 113 sorghum genotypes (S1 Table) from Hu et al. 2012 [21] was used to perform the phylogenetic analysis and PCA. An unrooted archaeopteryx tree was generated using the neighbor-joining cladogram function in the Trait Analysis by the Association Evolution and Linkage (TASSEL 5) program [56]. PCA was carried out using the snpgdsPCA function in the SNPRelate package in R [57]. The PCA visualization was created in ggplot2 [58].

### *Xvh* genome sequencing, assembly and annotation

The draft genome of *X*. *vasicola* pv. *holcicola* BLS185 was generated at the DOE Joint Genome Institute (JGI) using Pacific Biosciences (PacBio) sequencing technology [59] and is publicly available (https://img.jgi.doe.gov/cgi-bin/m/main.cgi?section=TaxonDetail&page=taxonDetail&taxon_oid=2757320517). To prepare the sample for sequencing, bacterial samples were grown on plates containing NYGA medium for 4 days at room temperature (approximately 22°C). Next, two, 5 ml cultures (NYG media, 15 ml culture tubes) were grown overnight with shaking (30°C). These cultures were combined, and genomic DNA was isolated using a standard CTAB-based protocol. For sequencing, a PacBio SMRTbell library was constructed and sequenced on the PacBio RS platform, which generated 163,972 filtered subreads totaling 903.8 Mbp. The raw reads were assembled using HGAP (version: 2.3.0 p5, protocol version = 2.3.0 method = RS HGAP Assembly.3,smrtpipe.py v1.87.139483,) [60]. The final draft assembly contained 2 contigs in 2 scaffolds, totaling 5.040 Mbp in size. The input read coverage was 98.6X. For annotation, genes were identified using Prodigal [61], followed by a round of manual curation using GenePRIMP [62]. The predicted CDSs were translated and used to search the National Center for Biotechnology Information (NCBI) non-redundant database, UniProt, TIGRFam, Pfam, KEGG, COG, and InterPro databases. The tRNAScanSE tool [63] was used to find tRNA genes, whereas ribosomal RNA genes were found with searches against models of the ribosomal RNA genes built from SILVA [64]. Other non–coding RNAs such as the RNA components of the protein secretion complex and RNase P were identified by searching the genome for the corresponding Rfam profiles using INFERNAL (Inference of RNA alignments, http://infernal.janelia.org.). Additional gene prediction analysis and manual functional annotation were performed within the Integrated Microbial Genomes (IMG) platform (http://img.jgi.doe.gov.) developed by the JGI, Walnut Creek, CA, USA [65].

## Supporting information

S1 Fig *Xvh* disease phenotypes of 156 sorghum genotypes at 7 dpi. Sorghum leaves were infiltrated with *Xvh* at OD600nm = 0.02 (~1 × 10^7^ cfu/mL). Green, red, and blue colors of ID text represent water-soaked lesions, red lesions, and resistance phenotypes, respectively.
(PDF)

S2 Fig Comparison of bacterial growth for *Xvh* and a non-host pathogen in Black Spanish (BS), Grassl, and NTJ2. Bacteria were infiltrated into sorghum leaves at an OD_600nm_ = 0.02 (~1 × 10^7^ cfu/mL). Bacterial populations (colony forming units, CFU) were determined from leaves 0 days and 7 days post-infiltration (dpi). Infected with *Xvh*, sorghum genotypes BS, Grassl, and NTJ2 displayed water-soaked lesions, red lesions, and resistance phenotypes, respectively. *X*. *axonopodis* pv. *manihotis Xam668* causes disease on cassava, not sorghum. Asterisks indicate statistical significance based on unequal variances *t* test (n = 3, *p < 0.05) of pairwise comparisons. Each replicate represents two inoculation areas from one leaf on one plant.
(PDF)

S3 Fig Distribution of *Xvh* disease phenotypes across a phylogenetic tree. There is no significant correlation between *Xvh* disease phenotypes and phylogeny. The unrooted neighbor-joining tree was constructed with GBS-based SNPs data from 113 sorghum genotypes. PI642998 = Black Spanish. Black Spanish (water-soaked lesions), Grassl (red lesions), and NTJ2 (resistance).
(PDF)

S4 Fig *Xvh* disease symptoms and bacterial growth in Black Spanish (BS), Grassl, and NTJ2 leaves with high titer inoculum at 48 hpi. (A) Disease symptoms on sorghum leaves from three genotypes (BS, Grassl, and NTJ2) at 48 hpi. (B) Bacterial populations were determined at 48 hpi and are shown as colony-forming units (CFU). Sorghum leaves were infiltrated with *Xvh* (OD_600nm_ = 0.5 (~1 × 10^9^ cfu/mL)). Mean ± s.d.; n = 3 biological replicates. Each replicate represents two inoculation areas from one leaf on one plant. hpi, hours post-inoculation.
(PDF)

S5 Fig Gene Ontology (GO)-term enrichment analysis for the DEGs (FDR adjusted *p* < 0.05; |log_2_ fold change| > 2) of sorghum (A) and *Xvh* (B) in different comparisons. Top 5 GO terms based on *P* values (adjusted by the Benjamini-Hochberg method) are listed in this figure. *P* values follow each GO term. Different comparisons in Venn diagram (Fig 6) and GO analysis are shown indicated in matching colored patterns. Bold text indicates GO terms relevant to host-pathogen interactions. Complete enriched GO terms are provided in S6 and S8 Tables. BS (water-soaked lesions), Grassl (red lesions), and NTJ2 (resistance).
(PDF)

S6 Fig Generation and evaluation of *XvhΔhrcC*. (A) Schematic representation of the genomic region of *hrcC* in *Xvh* and *XvhΔhrcC*. *XvhΔhrcC* was generated by the allelic exchange method. The deletion mutation is in-frame (3n = 24 nt) and confirmed by PCR and sequencing with primers Pf and Pr. M, DNA marker (ladder). (B) Disease symptoms on three sorghum genotypes (BS, Grassl, and NTJ2) at 7 dpi. (C) Bacterial populations shown as colony formation units (cfu) of cultures of bacteria recovered from sorghum leaves at 0 dpi and 7 dpi. Mean ± s.d.; n = 4 biological replicates. Sorghum leaves were infiltrated with *Xvh* (OD_600nm_ = 0.02 (~1 × 10^7^ cfu/mL)). Each replicate represents two inoculation areas from one leaf on one plant. Asterisks indicate statistical significance based on unequal variances *t* test (n = 4, *p < 0.05). BS (water-soaked lesions), Grassl (red lesions), and NTJ2 (resistance).
(PDF)

S7 Fig Expression patterns of *Xvh* and sorghum genes were validated by qRT-PCR. qRT-PCR results are reported as normalized relative quantities (NRQs) relative to sorghum *GAPDH* expression (A) or *Xvh gyrA* (B). (C-D) RNA-seq expression of *PR4* and *hrpF*. *Xvh*-infected sorghum genotypes BS, Grassl, and NTJ2 displayed water-soaked lesions, red lesions, and resistance phenotypes, respectively. hpi, hours post-inoculation. mean ± s.d.; n = 3 biological replicates. Each plant-mock or plant-*Xvh* replicate contained three inoculation areas from three leaves from three individual plants. Each *Xvh* in culture replicate contained ~1 × 10^8^ bacterial cells.
(PDF)

S8 Fig Heatmap showing expression levels of genes related to the T3SS and T3Es in *Pto* across *in vitro*, ETS, PTI, and ETI interactions. The comparison samples include: *Pto* in King’s B medium; *Pto* in T3SS/T3E-inducible minimal medium; *Pto*, *Pto* AvrRps4 (*Pto* strain ectopically expressing T3E AvrRps4), *Pto* AvrRpt2 (*Pto* strain ectopically expressing T3E AvrRpt2), and *Pto* D36E (*Pto* mutant strain lacking all 36 known *Pto* T3Es) infection in *Arabidopsis thaliana* Col-0 with ETS, ETI, ETI, and PTI phenotypes, respectively. The values represent the average of FPKM (fragments per kilobase per million mapped sequence reads) of all replicates for each condition. The raw sequencing data was generated by Nobori et al. 2018 [25].
(PDF)

S9 Fig Expression of T3SS-associated gene in apoplastic fluid from sorghum leaves. (A) *Xvh* expressing *hrpF* fused to a luciferase (Luc) reporter [*Xvh* (*hrpF*_promoter_*Luc*)] (OD_600nm_ = 0.6) was grown in T3SS/T3E-inducible minimal media (MM) mixed with apoplastic fluid collected from BS and NTJ2, or with sterile Milli-Q water (control). Bacteria were cultured for 12 hours and then adjusted to OD_600nm_ = 0.6 for assaying luciferase activity. (B) Bacterial populations (OD_600nm_ = 0.6) were determined and are shown as colony-forming units (CFU). Mean ± s.d.; n = 4 biological replicates.
(PDF)

S10 Fig Effects of amino acids on the expression of *hrpF* of *Xvh*. (A-B) *In vitro* assay. *Xvh* expressing *hrpF* fused to a luciferase (Luc) reporter [*Xvh* (*hrpF*_promoter_*Luc*)] grown in T3SS/T3E-inducible minimal media (MM) in absence or presence of the indicated nutrients. Peptone was used at 2% (w/v). Cysteine (Cys) and methionine (Met) were used at 0.06% (w/v), since the concentration of amino nitrogen in peptone is equal to or greater than 3% (HiMedia Laboratories). Bacteria were cultured for 12 hours and then adjusted to OD_600nm_ = 0.6 for assaying luciferase activity (A). (B) Bacterial populations at OD_600nm_ = 0.6. CFU, colony-forming units. Mean ± s.d.; n = 4 biological replicates. Asterisks indicate statistical significance based on unequal variances *t* test (n = 4, *p < 0.05) comparison with MM treatment. (C-D) *In planta* assay. Sorghum NTJ2 leaves were infected with *Xvh* (*hrpF*_promoter_*Luc*) (OD_600nm_ = 0.5 (~1 × 10^9^ cfu/mL)) in the absence or presence of the indicated nutrients. Luciferase activity assay was performed at 48 hpi (C). Peptone, Cys, and Met were used at 0.5% (w/v), 0.0015% (w/v), and 0.0015% (w/v), respectively. (D) Bacterial populations in sorghum were quantified at 48 hpi. Mean ± s.d.; n = 4 biological replicates. Asterisks indicate statistical significance based on unequal variances *t* test (n = 4, *p < 0.05) comparison with *Xvh* infection.
(PDF)

S11 Fig Snapshot of the continually evolving host-pathogen arms race. The original zig-zag model describes the relative strength of defense response across the spectrum of pathogen-induced plant phenotypes (Jones and Dangl 2006). Stronger plant defense response (black line) equates with increased resistance. Here, a new dimension, strength of pathogen-virulence response, is proposed. As one of multiple snapshots of the continually evolving host-pathogen arms race, an increased virulence response (red line) corresponds to a plant resistance response. PRRs: pattern recognition receptors, T3Es: type III effectors, R: plant resistance genes recognize, directly or indirectly, a pathogen effector.
(PDF)

S1 Table Summary of 156 sorghum genotypes. (XLSX)

S2 Table Summary of dual RNA-seq libraries and sequencing. (XLSX)

S3 Table Summary of Venn diagram analysis for differentially expressed genes (FDR adjusted p < 0.05; |log2 fold change| > 2) of sorghum. (XLSX)

S4 Table Summary of Venn diagram analysis for differentially expressed genes (FDR adjusted p < 0.05; |log2 fold change| > 2) of *Xvh*. (XLSX)

S5 Table Differentially expressed genes (FDR adjusted p < 0.05; |log2 fold change| > 2) of sorghum for GO-term enrichment analysis. (XLSX)

S6 Table Summary of GO-term enrichment analysis for the differentially expressed genes (FDR adjusted p < 0.05; |log2 fold change| > 2) of sorghum. (XLSX)

S7 Table Differentially expressed genes (FDR adjusted p < 0.05; |log2 fold change| > 2) of *Xvh* for GO-term enrichment analysis. (XLSX)

S8 Table Summary of GO-term enrichment analysis for the differentially expressed genes (FDR adjusted p < 0.05; |log2 fold change| > 2) of *Xvh*. (XLSX)

S9 Table Summary of GO-term enrichment analysis for uniquely differentially expressed genes of *Xvh* in BS-*Xvh*, Grassl-*Xvh*, and NTJ2-*Xvh*, respectively. (XLSX)

S10 Table Summary of GO-term enrichment analysis for uniquely differentially expressed genes of sorghum in BS-*Xvh*, Grassl-*Xvh*, and NTJ2-*Xvh*, respectively. (XLSX)

S11 Table Summary of hierarchical clustering analysis based on gene expression from mock and *Xvh*-infected sorghum samples. (XLSX)

S12 Table Summary of hierarchical clustering analysis based on gene expression from *Xvh* culture and *in planta* samples. (XLSX)

S13 Table Primers used in this study. (XLSX)
