# Supplementary material for: Escalation in the host-pathogen arms race: A host resistance response corresponds to a heightened bacterial virulence response
Source: PLoS Pathog. 2021 Jan 11;17(1):e1009175. doi: 10.1371/journal.ppat.1009175 (PMC7822516; doi:10.1371/journal.ppat.1009175)
Supplement: S1 Fig — Sorghum leaves were infiltrated with Xvh at OD600nm = 0.02 (~1 × 10^7^ cfu/mL). Green, red, and blue colors of ID text represent water-soaked lesions, red lesions, and resistance phenotypes, respectively. (PDF) [file ppat.1009175.s001.pdf]

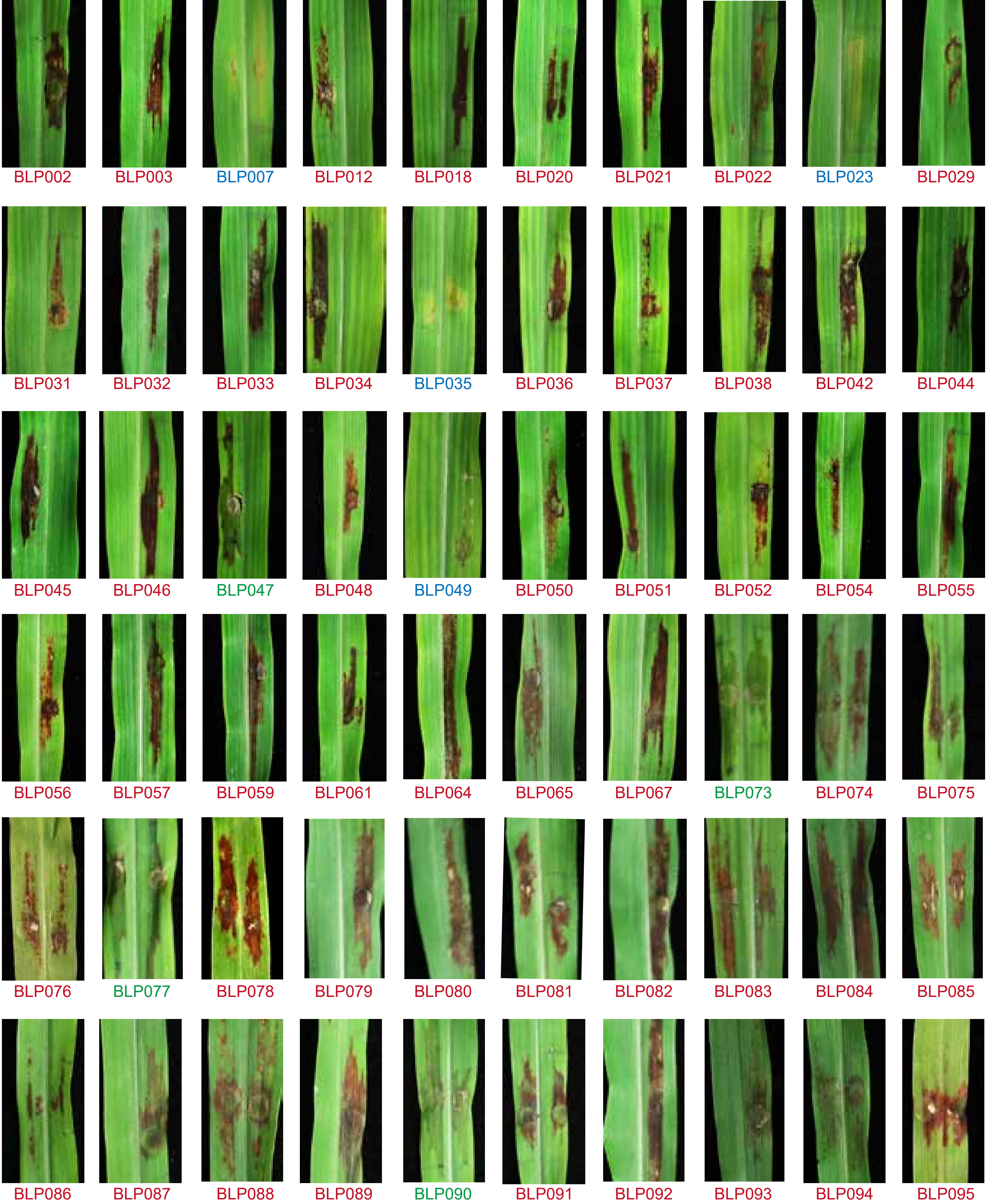
BLP002
BLP003
BLP007
BLP012
BLP018
BLP020
BLP021
BLP022
BLP023
BLP029
BLP031
BLP032
BLP033
BLP034
BLP035
BLP036
BLP037
BLP038
BLP042
BLP044
BLP045
BLP046
BLP047
BLP048
BLP049
BLP050
BLP051
BLP052
BLP054
BLP055
BLP056
BLP057
BLP059
BLP061
BLP064
BLP065
BLP067
BLP073
BLP074
BLP075
BLP076
BLP077
BLP078
BLP079
BLP080
BLP081
BLP082
BLP083
BLP084
BLP085
BLP086
BLP087
BLP088
BLP089
BLP090
BLP091
BLP092
BLP093
BLP094
BLP095

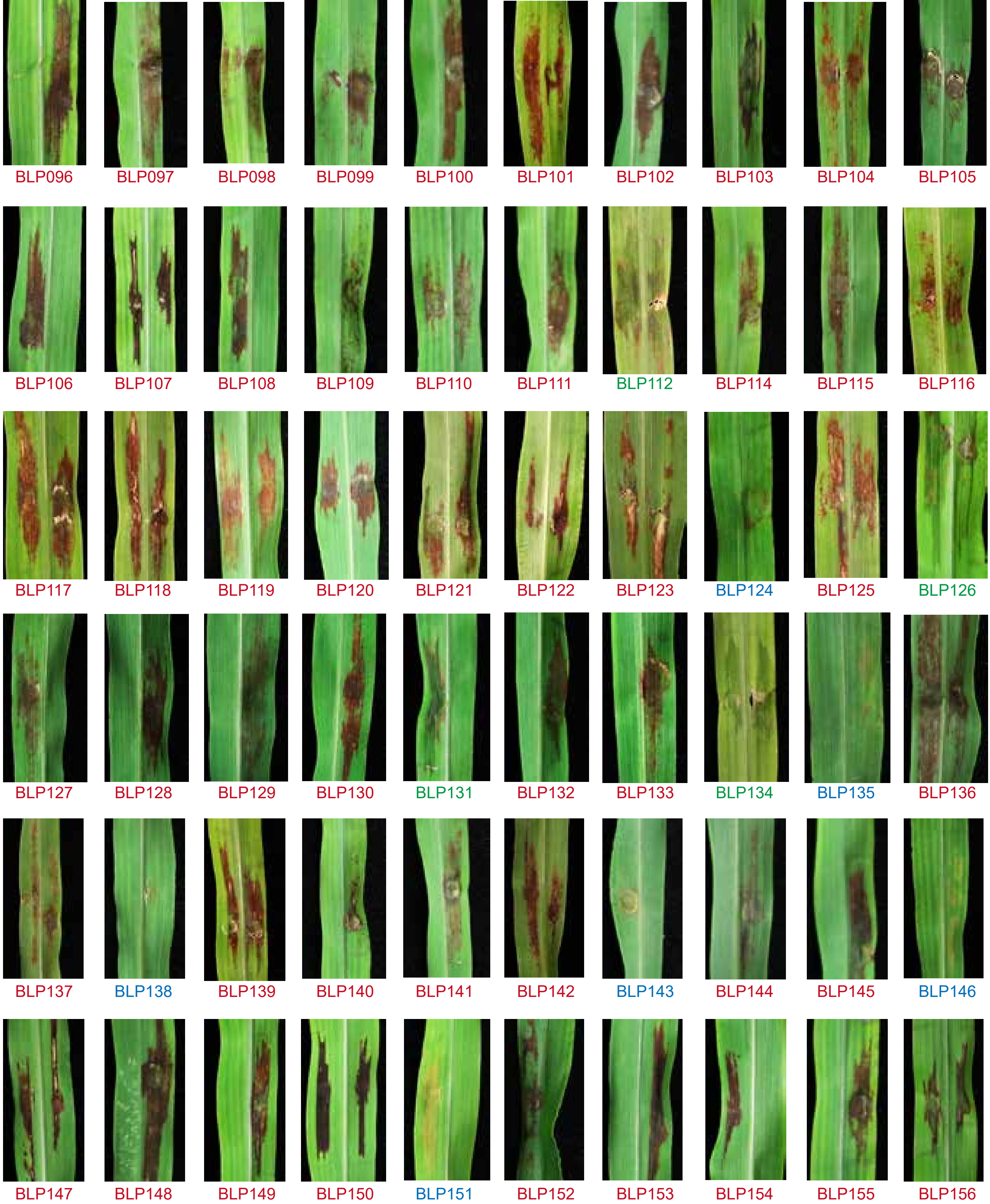
BLP096
BLP097
BLP098
BLP099
BLP100
BLP101
BLP102
BLP103
BLP104
BLP105
BLP106
BLP107
BLP108
BLP109
BLP110
BLP111
BLP112
BLP114
BLP115
BLP116
BLP117
BLP118
BLP119
BLP120
BLP121
BLP122
BLP123
BLP124
BLP125
BLP126
BLP127
BLP128
BLP129
BLP130
BLP131
BLP132
BLP133
BLP134
BLP135
BLP136
BLP137
BLP138
BLP139
BLP140
BLP141
BLP142
BLP143
BLP144
BLP145
BLP146
BLP147
BLP148
BLP149
BLP150
BLP151
BLP152
BLP153
BLP154
BLP155
BLP156

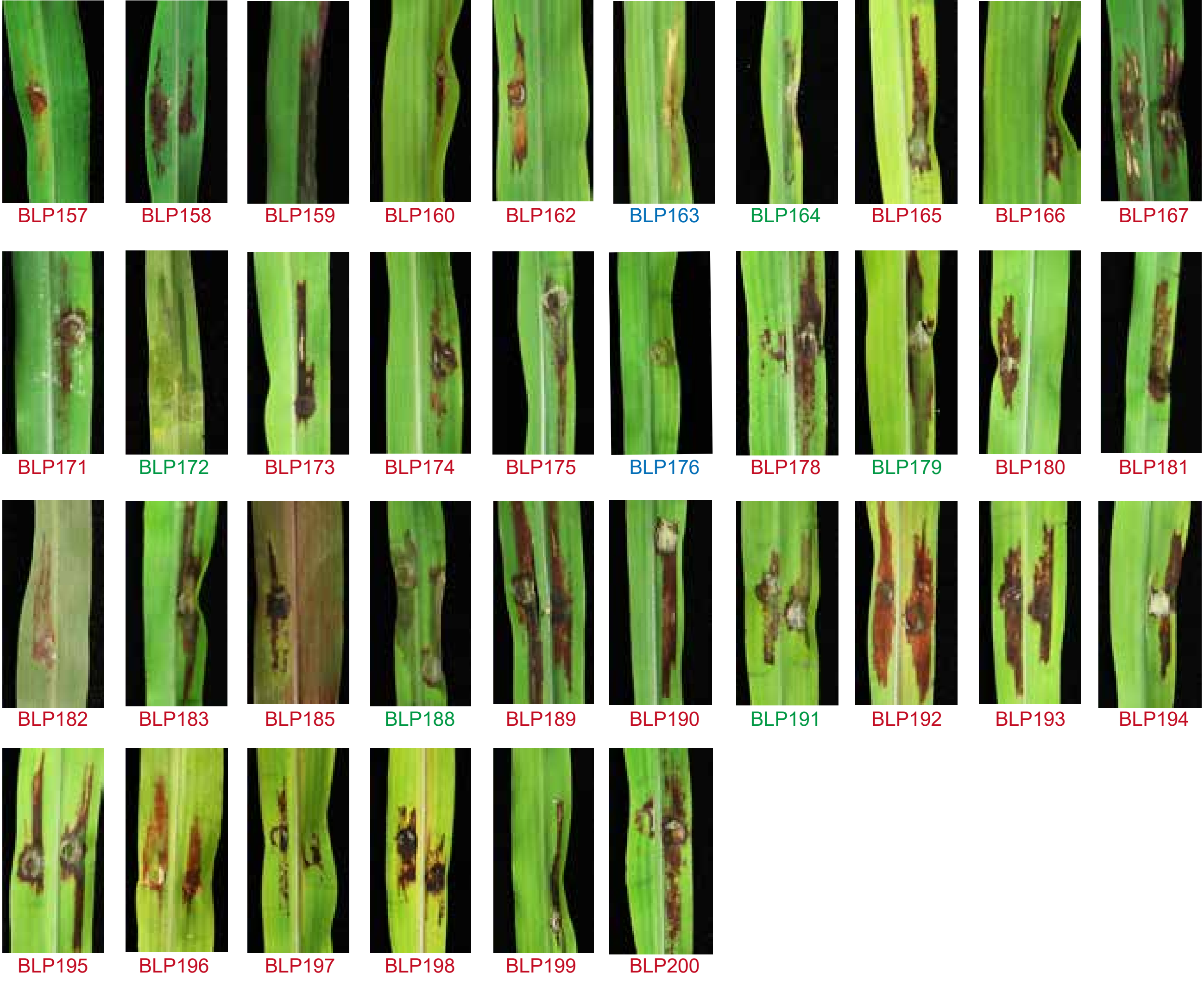
BLP157
BLP158
BLP159
BLP160
BLP162
BLP163
BLP164
BLP165
BLP166
BLP167
BLP171
BLP172
BLP173
BLP174
BLP175
BLP176
BLP178
BLP179
BLP180
BLP181
BLP182
BLP183
BLP185
BLP188
BLP189
BLP190
BLP191
BLP192
BLP193
BLP194
BLP195
BLP196
BLP197
BLP198
BLP199
BLP200
