# Supplementary figures and images for: Escalation in the host-pathogen arms race: A host resistance response corresponds to a heightened bacterial virulence response
Source: PLoS Pathog. 2021 Jan 11;17(1):e1009175. doi: 10.1371/journal.ppat.1009175 (PMC7822516; doi:10.1371/journal.ppat.1009175)

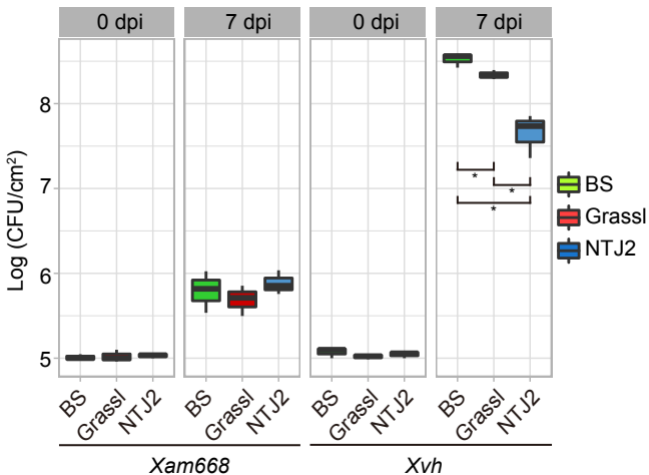

Supplement: S2 Fig — Bacteria were infiltrated into sorghum leaves at an OD600nm = 0.02 (~1 × 10^7^ cfu/mL). Bacterial populations (colony forming units, CFU) were determined from leaves 0 days and 7 days post-infiltration (dpi). Infected with Xvh, sorghum genotypes BS, Grassl, and NTJ2 displayed water-soaked lesions, red lesions, and resistance phenotypes, respectively. X. axonopodis pv. manihotis Xam668 causes disease on cassava, not sorghum. Asterisks indicate statistical significance based on unequal variances t test (n = 3, *p < 0.05) of pairwise comparisons. Each replicate represents two inoculation areas from one leaf on one plant. (PDF) [file ppat.1009175.s002.pdf]

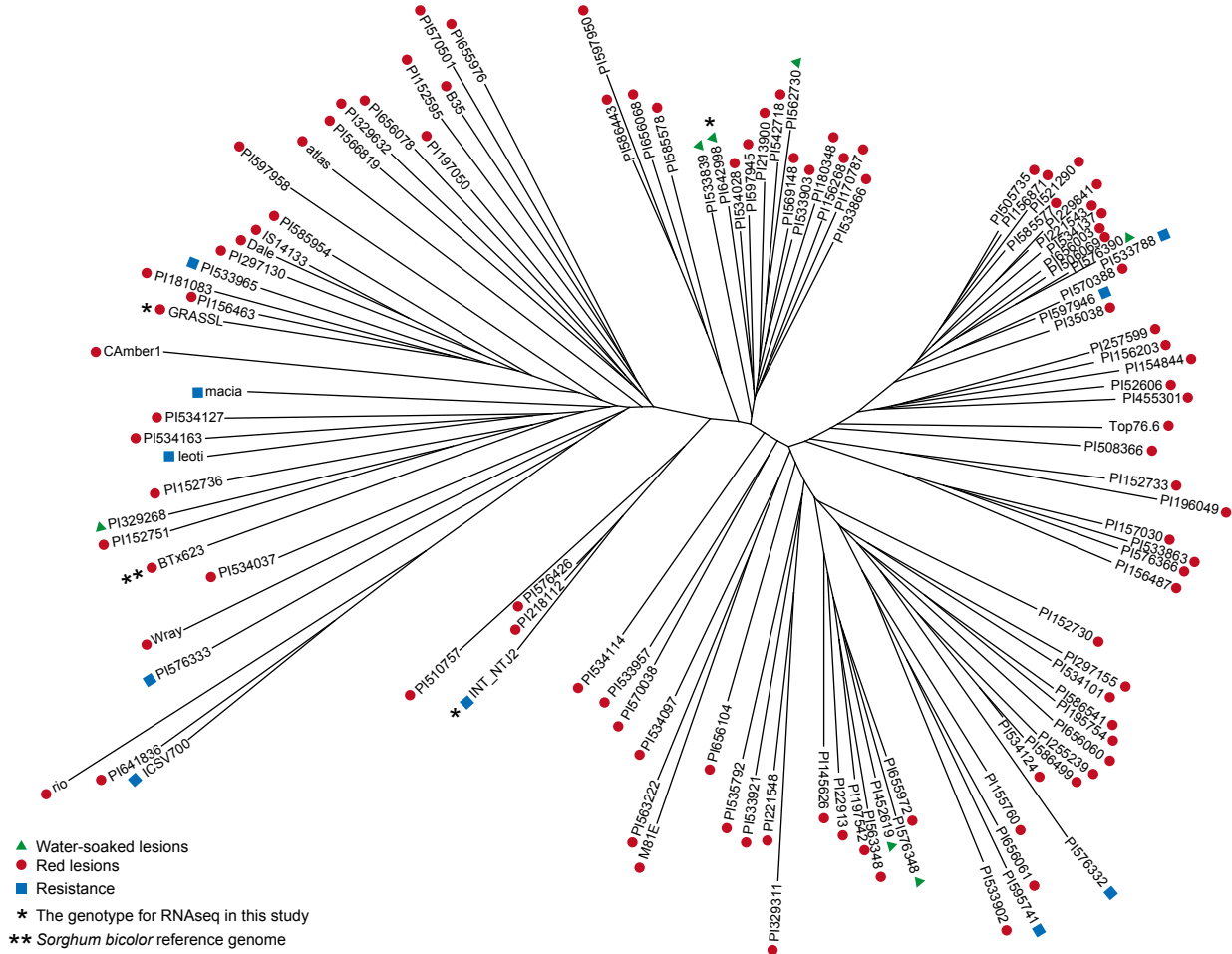

Supplement: S3 Fig — There is no significant correlation between Xvh disease phenotypes and phylogeny. The unrooted neighbor-joining tree was constructed with GBS-based SNPs data from 113 sorghum genotypes. PI642998 = Black Spanish. Black Spanish (water-soaked lesions), Grassl (red lesions), and NTJ2 (resistance). (PDF) [file ppat.1009175.s003.pdf]

A

48 hpi

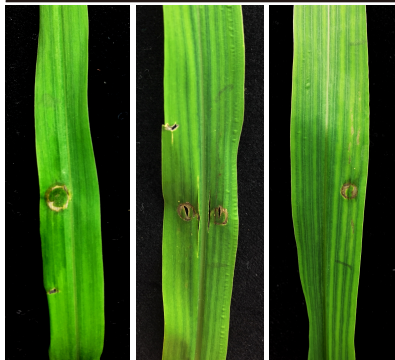

BS

Grassl

NTJ2

B

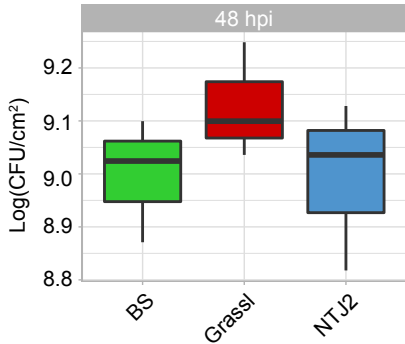

Supplement: S4 Fig — (A) Disease symptoms on sorghum leaves from three genotypes (BS, Grassl, and NTJ2) at 48 hpi. (B) Bacterial populations were determined at 48 hpi and are shown as colony-forming units (CFU). Sorghum leaves were infiltrated with Xvh (OD600nm = 0.5 (~1 × 10^9^ cfu/mL)). Mean ± s.d.; n = 3 biological replicates. Each replicate represents two inoculation areas from one leaf on one plant. hpi, hours post-inoculation. (PDF) [file ppat.1009175.s004.pdf]

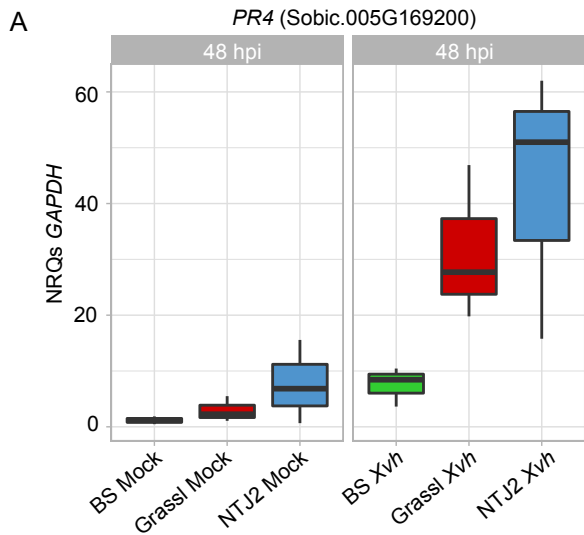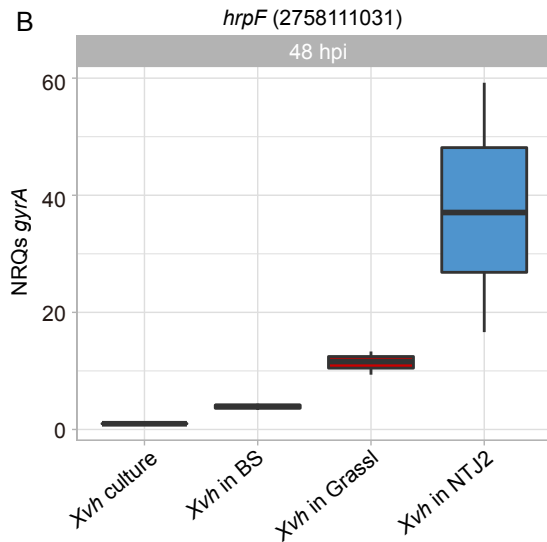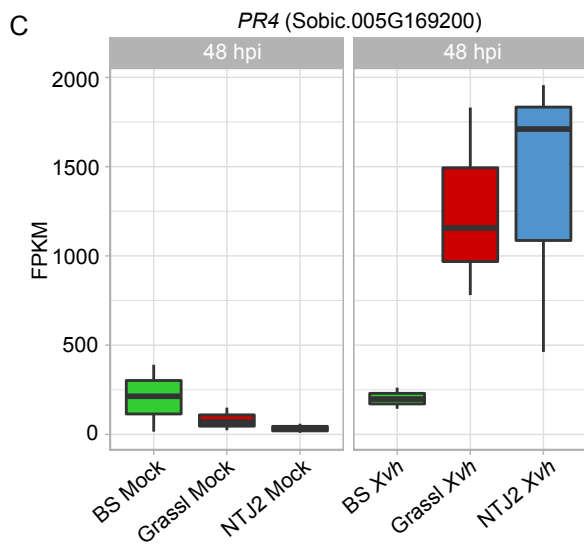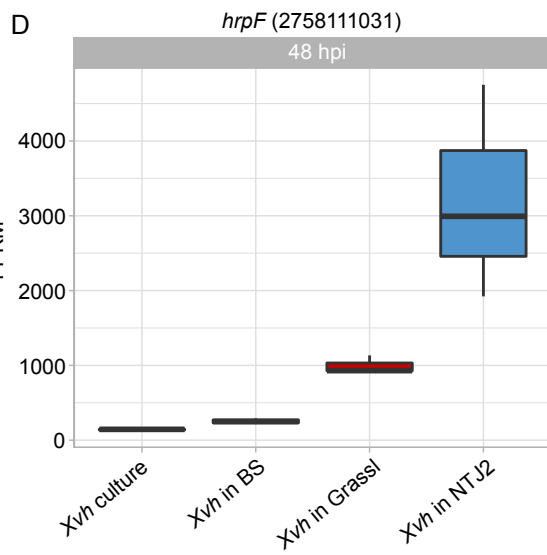

Supplement: S7 Fig — qRT-PCR results are reported as normalized relative quantities (NRQs) relative to sorghum GAPDH expression (A) or Xvh gyrA (B). (C-D) RNA-seq expression of PR4 and hrpF. Xvh-infected sorghum genotypes BS, Grassl, and NTJ2 displayed water-soaked lesions, red lesions, and resistance phenotypes, respectively. hpi, hours post-inoculation. mean ± s.d.; n = 3 biological replicates. Each plant-mock or plant-Xvh replicate contained three inoculation areas from three leaves from three individual plants. Each Xvh in culture replicate contained ~1 × 10^8^ bacterial cells. (PDF) [file ppat.1009175.s007.pdf]

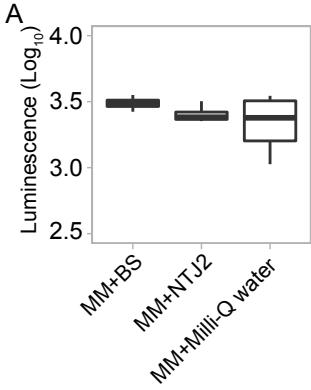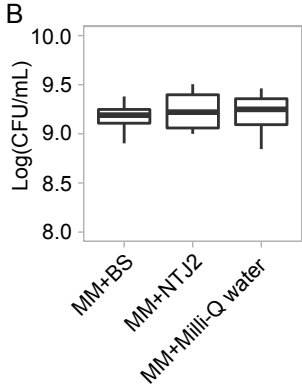

Supplement: S9 Fig — (A) Xvh expressing hrpF fused to a luciferase (Luc) reporter [Xvh (hrpF_promoter_Luc)] (OD600nm = 0.6) was grown in T3SS/T3E-inducible minimal media (MM) mixed with apoplastic fluid collected from BS and NTJ2, or with sterile Milli-Q water (control). Bacteria were cultured for 12 hours and then adjusted to OD600nm = 0.6 for assaying luciferase activity. (B) Bacterial populations (OD600nm = 0.6) were determined and are shown as colony-forming units (CFU). Mean ± s.d.; n = 4 biological replicates. (PDF) [file ppat.1009175.s009.pdf]

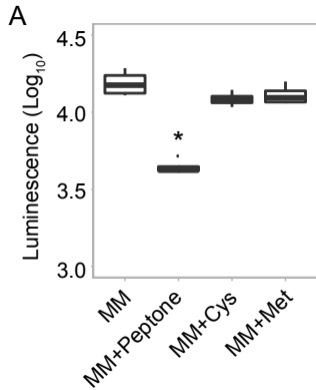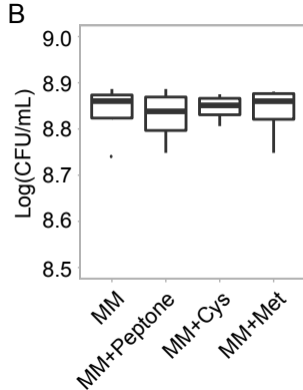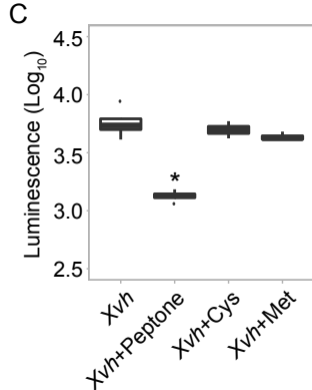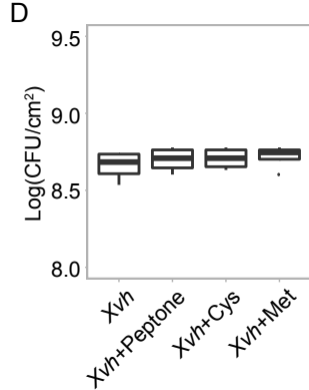

Supplement: S10 Fig — (A-B) In vitro assay. Xvh expressing hrpF fused to a luciferase (Luc) reporter [Xvh (hrpF_promoter_Luc)] grown in T3SS/T3E-inducible minimal media (MM) in absence or presence of the indicated nutrients. Peptone was used at 2% (w/v). Cysteine (Cys) and methionine (Met) were used at 0.06% (w/v), since the concentration of amino nitrogen in peptone is equal to or greater than 3% (HiMedia Laboratories). Bacteria were cultured for 12 hours and then adjusted to OD600nm = 0.6 for assaying luciferase activity (A). (B) Bacterial populations at OD600nm = 0.6. CFU, colony-forming units. Mean ± s.d.; n = 4 biological replicates. Asterisks indicate statistical significance based on unequal variances t test (n = 4, *p < 0.05) comparison with MM treatment. (C-D) In planta assay. Sorghum NTJ2 leaves were infected with Xvh (hrpF_promoter_Luc) (OD600nm = 0.5 (~1 × 10^9^ cfu/mL)) in the absence or presence of the indicated nutrients. Luciferase activity assay was performed at 48 hpi (C). Peptone, Cys, and Met were used at 0.5% (w/v), 0.0015% (w/v), and 0.0015% (w/v), respectively. (D) Bacterial populations in sorghum were quantified at 48 hpi. Mean ± s.d.; n = 4 biological replicates. Asterisks indicate statistical significance based on unequal variances t test (n = 4, *p < 0.05) comparison with Xvh infection. (PDF) [file ppat.1009175.s010.pdf]

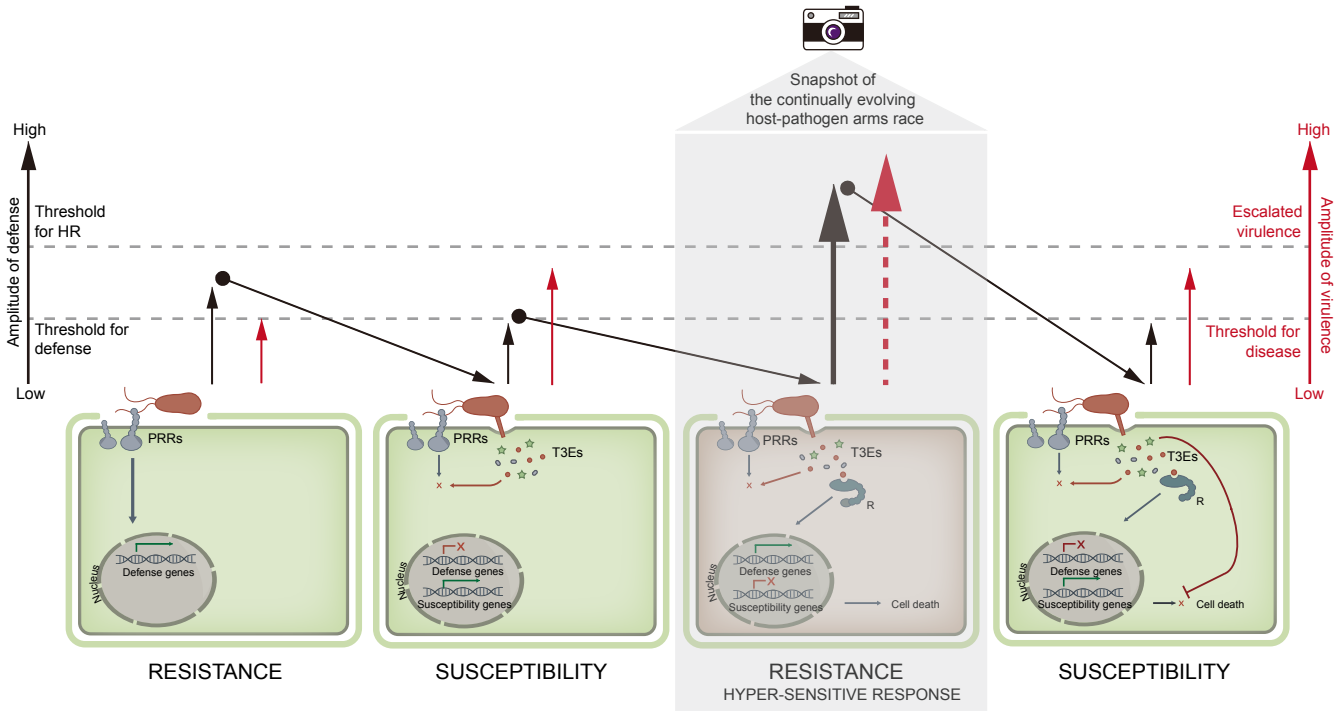

Supplement: S11 Fig — The original zig-zag model describes the relative strength of defense response across the spectrum of pathogen-induced plant phenotypes (Jones and Dangl 2006). Stronger plant defense response (black line) equates with increased resistance. Here, a new dimension, strength of pathogen-virulence response, is proposed. As one of multiple snapshots of the continually evolving host-pathogen arms race, an increased virulence response (red line) corresponds to a plant resistance response. PRRs: pattern recognition receptors, T3Es: type III effectors, R: plant resistance genes recognize, directly or indirectly, a pathogen effector. (PDF) [file ppat.1009175.s011.pdf]
