# Supplementary material for: Escalation in the host-pathogen arms race: A host resistance response corresponds to a heightened bacterial virulence response
Source: PLoS Pathog. 2021 Jan 11;17(1):e1009175. doi: 10.1371/journal.ppat.1009175 (PMC7822516; doi:10.1371/journal.ppat.1009175)
Supplement: S5 Fig — Top 5 GO terms based on P values (adjusted by the Benjamini-Hochberg method) are listed in this figure. P values follow each GO term. Different comparisons in Venn diagram (Fig 6) and GO analysis are shown indicated in matching colored patterns. Bold text indicates GO terms relevant to host-pathogen interactions. Complete enriched GO terms are provided in S6 and S8 Tables. BS (water-soaked lesions), Grassl (red lesions), and NTJ2 (resistance). (PDF) [file ppat.1009175.s005.pdf]

A

	BS-*Xvh* vs BS-mock	Grassl-*Xvh* vs Grassl-mock	NTJ2-*Xvh* vs NTJ2-mock
Induced (GO)	Sequence-specific DNA binding (1.03E-9)	Protein amino acid phosphorylation (1.41E-15)	Protein amino acid phosphorylation (4.23E-17)
	Post-translational protein modification (6.54E-9)	Protein kinase activity (3.74E-15)	Protein kinase activity (9.44E-17)
	Protein modification process (1.35E-8)	Post-translational protein modification (1.87E-14)	Phosphorylation (7.42E-16)
	Macromolecule modification (3.98E-8)	Phosphorylation (4.51E-14)	Carbohydrate binding (6.51E-15)
	Protein amino acid phosphorylation (4.22E-8)	Protein modification process (6.64E-14)	Protein modification process (1.96E-14)
Suppressed (GO)	No GO enriched	Oxygen evolving complex (7.94E-08)	Transcription regulator activity (1.23E-08)
		Extrinsic to membrane (1.56E-07)	Transcription factor activity (7.02E-08)
		Photosystem II (1.27E-05)	Iron ion binding (4.74E-06)
		Photosynthesis (3.57E-05)	Regulation of transcription, DNA-dependent (5.26E-05)
		Protein folding (5.49E-05)	Regulation of RNA metabolic process (5.37E-05)

B

	Xvh in BS vs *Xvh* culture	*Xvh* in Grassl vs *Xvh* culture	*Xvh* in NTJ2 vs *Xvh* culture
Induced (GO)	Structural constituent of ribosome (1.67E-11)	Macromolecular complex (4.63E-09)	**Protein secretion by the type III secretion system** (9.75E-09)
	Cellular amino acid biosynthetic process (1.75E-11)	Structural constituent of ribosome (5.34E-09)	**Type III protein secretion system complex** (2.72E-06)
	Ribosome (2.65E-11)	Cytoplasmic part (7.66E-09)	Establishment of localization (1.62E-04)
	Ribonucleoprotein complex (4.15E-11)	Ribosome (7.90E-09)	Transport (1.62E-04)
	Amine biosynthetic process (7.77E-11)	Ribonucleoprotein complex (1.16E-08)	Potassium ion-transporting ATPase complex (4.66E-04)
Suppressed (GO)	Protein folding (8.69E-07)	Transition metal ion binding (1.29E-05)	Molecular transducer activity (1.26E-05)
	Unfolded protein binding (1.38E-05)	Metal ion binding (2.80E-04)	Signal transducer activity (1.26E-05)
		Cation binding (5.61E-04)	Signaling process (5.76E-05)
		Double-strand break repair (DSBR) (7.14E-04)	Signal transmission (5.76E-05)
		DSBR via nonhomologous end joining (7.14E-04)	Signaling (6.62E-05)
