# Supplementary material for: Escalation in the host-pathogen arms race: A host resistance response corresponds to a heightened bacterial virulence response
Source: PLoS Pathog. 2021 Jan 11;17(1):e1009175. doi: 10.1371/journal.ppat.1009175 (PMC7822516; doi:10.1371/journal.ppat.1009175)

Gene	Pto_King's B	Pto_Minimal medium	Pto_ETS	Pto AvrRps4_ETI	Pto AvrRpt2_ETI	Pto D36E_PTI	Annotation
PSPTO_0044	223	131	211	200	212	0	type III effector HopK1
PSPTO_0061	343	559	740	883	508	810	type III effector HopY1
PSPTO_0474	0	1	7	0	3	0	type III effector HopAS1
PSPTO_0501	29	25	30	23	30	0	type III effector HopU1
PSPTO_0502	56	101	83	118	62	8	type III effector HopF2
PSPTO_0503	143	826	330	830	265	2325	type III chaperone protein ShcF
PSPTO_0588	430	862	556	772	411	0	type III effector HopH1
PSPTO_0589	127	761	276	257	188	0	type III effector HopC1
PSPTO_0852	51	186	76	97	51	122	type III helper protein HopAJ1
PSPTO_0876	147	70	167	154	130	0	type III effector HopD1
PSPTO_0877	65	64	82	94	68	0	type III effector HopQ1-1
PSPTO_0883	25	21	29	19	24	0	type III effector HopR1
PSPTO_0901	52	21	28	12	23	21	type III effector HopAG1
PSPTO_0905	8	10	5	6	4	13	type III effector HopAH1
PSPTO_0906	12	12	22	33	18	12	type III effector HopAI1
PSPTO_1022	75	857	141	260	98	0	type III effector HopAM1-1
PSPTO_1369	265	1222	731	813	475	0	type III chaperone protein ShcN
PSPTO_1370	69	73	61	65	69	0	type III effector HopN1
PSPTO_1372	9	33	34	65	27	0	type III effector HopAA1-1
PSPTO_1373	89	491	210	220	122	0	type III helper protein HrpW1
PSPTO_1374	168	766	279	434	211	0	type III chaperone ShcM
PSPTO_1375	30	107	66	100	45	0	type III effector HopM1
PSPTO_1376	68	236	127	211	115	0	type III chaperone ShcE
PSPTO_1377	16	56	23	20	17	0	type III effector protein AvrE1
PSPTO_1379	32	49	54	100	38	71	type III transcriptional regulator HrpR
PSPTO_1380	148	38	96	117	60	244	type III transcriptional regulator HrpS
PSPTO_1381	2189	12633	3360	6276	2240	8670	type III helper protein HrpA1
PSPTO_1382	138	316	301	656	211	436	type III restriction system endonuclease
PSPTO_1383	17	193	304	480	197	461	type III secretion protein HrpB
PSPTO_1384	149	379	308	433	197	283	type III secretion protein HrcJ
PSPTO_1385	38	348	337	519	244	449	type III secretion protein HrpD
PSPTO_1386	69	354	226	277	147	283	type III secretion protein HrpE
PSPTO_1387	18	1201	500	1277	435	1861	type III secretion protein HrpF
PSPTO_1388	275	848	653	987	481	1024	type III secretion protein HrpG
PSPTO_1389	57	237	188	298	129	189	outer-membrane type III secretion protein HrcC
PSPTO_1390	9	1058	549	657	456	948	type III secretion protein
PSPTO_1392	31	102	58	95	37	49	type III secretion protein HrcU
PSPTO_1393	10	68	38	61	25	45	type III secretion protein HrcT
PSPTO_1394	4	62	33	46	25	22	type III secretion protein HrcS
PSPTO_1395	57	161	148	260	103	191	type III secretion protein HrcR
PSPTO_1396	57	226	107	191	79	137	type III secretion protein HrcQb
PSPTO_1397	30	202	101	178	74	102	type III secretion protein HrcQa
PSPTO_1398	329	645	303	392	149	277	type III secretion protein HrpP
PSPTO_1399	276	1642	745	1584	504	1202	type III secretion protein HrpO
PSPTO_1400	25	126	53	98	38	135	type III secretion cytoplasmic ATPase HrcN
PSPTO_1401	55	405	179	374	116	450	type III secretion protein HrpQ
PSPTO_1402	46	6	32	67	34	127	type III secretion protein HrcV
PSPTO_1403	68	586	173	273	106	320	type III secretion protein HrpJ
PSPTO_1405	168	399	409	657	285	729	type III helper protein HrpK1
PSPTO_1406	31	161	106	144	69	0	type III effector HopB1
PSPTO_1568	23	38	24	44	15	0	type III effector HopAF1
PSPTO_2678	6	41	23	24	16	48	type III helper protein HopP1
PSPTO_3087	88	216	216	509	139	0	type III effector HopAB2
PSPTO_3292	26	11	3	2	3	4	type III effector HopAH2-1
PSPTO_3293	25	30	20	19	14	20	type III effector HopAH2-2
PSPTO_4001	2215	4861	3949	6118	3293	2	type III effector protein AvrPto1
PSPTO_4101	150	390	296	502	196	426	type III helper protein HopAK1
PSPTO_4331	337	1663	430	841	259	0	type III effector HopE1
PSPTO_4588	40	106	50	67	40	0	type III effector HopS2
PSPTO_4589	127	305	72	66	50	0	type III chaperone ShcS2
PSPTO_4590	67	61	38	65	30	0	type III effector HopT2
PSPTO_4592	96	102	43	44	38	0	type III effector HopO1-3
PSPTO_4593	21	36	24	19	24	0	type III effector HopT1-2
PSPTO_4594	18	27	14	13	12	0	type III effector HopO1-2
PSPTO_4597	146	190	174	152	112	0	type III effector HopS1
PSPTO_4599	344	1348	636	725	433	0	type III chaperone ShcS1
PSPTO_4691	65	25	106	22	82	0	type III effector HopAD1
PSPTO_4703	924	638	893	1267	692	358	type III effector HopAQ1
PSPTO_4718	30	57	32	34	21	0	type III effector HopAA1-2
PSPTO_4720	239	122	378	104	211	0	type III effector HopV1
PSPTO_4721	162	157	301	82	171	0	type III chaperone ShcV
PSPTO_4722	1118	85	1490	357	825	2	type III effector HopAO1
PSPTO_4724	69	34	67	45	38	0	type III effector HopD
PSPTO_4727	23	77	21	13	16	0	type III effector HopG1
PSPTO_4732	2	2	2	4	3	3	type III effector HopQ1-2
PSPTO_4776	69	172	93	124	57	0	type III effector HopI1
PSPTO_5353	76	232	134	235	74	0	type III chaperone protein ShcA
PSPTO_5354	59	239	104	134	70	0	type III effector HopA1
PSPTOA0005	74	851	140	259	97	0	type III effector HopAM1-2
PSPTOA0017	0	0	0	0	0	0	type III chaperone ShcO1
PSPTOA0019	0	0	0	0	0	0	type III effector HopT1-1

FPKM

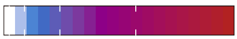

Supplement: S8 Fig — The comparison samples include: Pto in King’s B medium; Pto in T3SS/T3E-inducible minimal medium; Pto, Pto AvrRps4 (Pto strain ectopically expressing T3E AvrRps4), Pto AvrRpt2 (Pto strain ectopically expressing T3E AvrRpt2), and Pto D36E (Pto mutant strain lacking all 36 known Pto T3Es) infection in Arabidopsis thaliana Col-0 with ETS, ETI, ETI, and PTI phenotypes, respectively. The values represent the average of FPKM (fragments per kilobase per million mapped sequence reads) of all replicates for each condition. The raw sequencing data was generated by Nobori et al. 2018 [25]. (PDF) [file ppat.1009175.s008.pdf]
