# Supplementary material for: Escalation in the host-pathogen arms race: A host resistance response corresponds to a heightened bacterial virulence response
Source: PLoS Pathog. 2021 Jan 11;17(1):e1009175. doi: 10.1371/journal.ppat.1009175 (PMC7822516; doi:10.1371/journal.ppat.1009175)
Supplement: S6 Fig — (A) Schematic representation of the genomic region of hrcC in Xvh and XvhΔhrcC. XvhΔhrcC was generated by the allelic exchange method. The deletion mutation is in-frame (3n = 24 nt) and confirmed by PCR and sequencing with primers Pf and Pr. M, DNA marker (ladder). (B) Disease symptoms on three sorghum genotypes (BS, Grassl, and NTJ2) at 7 dpi. (C) Bacterial populations shown as colony formation units (cfu) of cultures of bacteria recovered from sorghum leaves at 0 dpi and 7 dpi. Mean ± s.d.; n = 4 biological replicates. Sorghum leaves were infiltrated with Xvh (OD600nm = 0.02 (~1 × 10^7^ cfu/mL)). Each replicate represents two inoculation areas from one leaf on one plant. Asterisks indicate statistical significance based on unequal variances t test (n = 4, *p < 0.05). BS (water-soaked lesions), Grassl (red lesions), and NTJ2 (resistance). (PDF) [file ppat.1009175.s006.pdf]

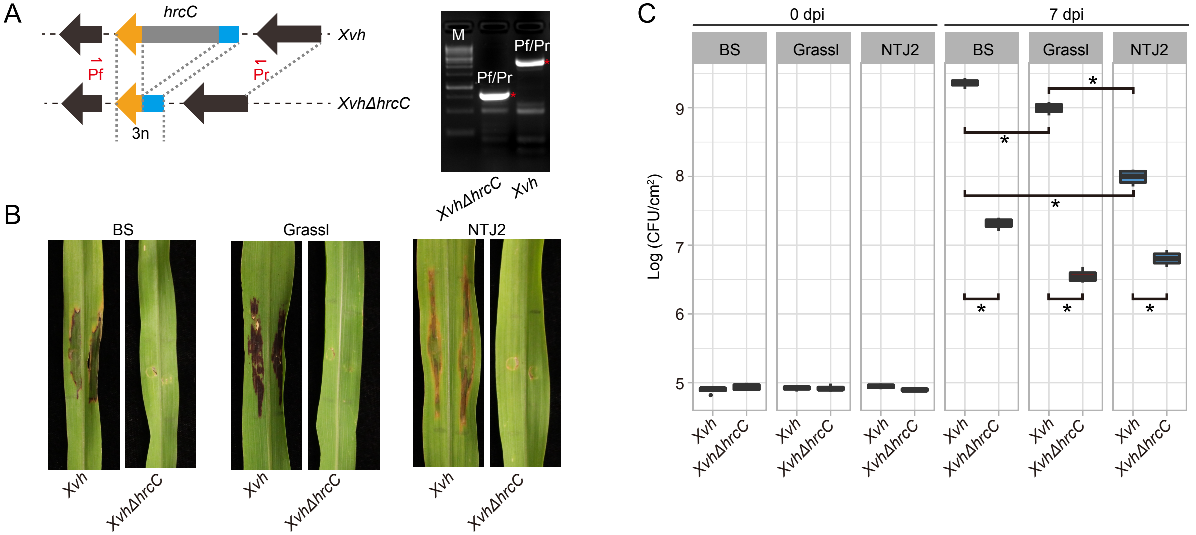

A
hrcC
Xvh
Pf
Pr
XvhΔhrcC
3n
M
Pf/Pr
Pf/Pr
XvhΔhrcC
Xvh
B
BS
Grassl
NTJ2
Xvh
XvhΔhrcC
Xvh
XvhΔhrcC
Xvh
XvhΔhrcC
C
0 dpi
7 dpi
BS
Grassl
NTJ2
BS
Grassl
NTJ2
Log (CFU/cm²)
9
8
7
6
5
Xvh
XvhΔhrcC
